# Conservation analysis of the CydX protein yields insights into small protein identification and evolution

**DOI:** 10.1186/1471-2164-15-946

**Published:** 2014-12-05

**Authors:** Rondine J Allen, Evan P Brenner, Caitlin E VanOrsdel, Jessica J Hobson, David J Hearn, Matthew R Hemm

**Affiliations:** Department of Biological Sciences, Towson University, Towson, 21252MD USA; College of Pharmacy, University of Iowa, Iowa City, 52242IA USA; University of Minnesota, Minneapolis, 55455MN USA

**Keywords:** CydX, Cytochrome *bd* oxidase, Small proteins, Phylogenetics, Small protein conservation

## Abstract

**Background:**

The reliable identification of proteins containing 50 or fewer amino acids is difficult due to the limited information content in short sequences. The 37 amino acid CydX protein in *Escherichia coli* is a member of the cytochrome *bd* oxidase complex, an enzyme found throughout Eubacteria. To investigate the extent of CydX conservation and prevalence and evaluate different methods of small protein homologue identification, we surveyed 1095 Eubacteria species for the presence of the small protein.

**Results:**

Over 300 homologues were identified, including 80 unannotated genes. The ability of both closely-related and divergent homologues to complement the *E. coli* Δ*cydX* mutant supports our identification techniques, and suggests that CydX homologues retain similar function among divergent species. However, sequence analysis of these proteins shows a great degree of variability, with only a few highly-conserved residues. An analysis of the co-variation between CydX homologues and their corresponding *cydA* and *cydB* genes shows a close synteny of the small protein with the CydA long Q-loop. Phylogenetic analysis suggests that the *cydABX* operon has undergone horizontal gene transfer, although the *cydX* gene likely evolved in a progenitor of the Alpha, Beta, and Gammaproteobacteria. Further investigation of *cydAB* operons identified two additional conserved hypothetical small proteins: CydY encoded in CydA_Qlong_ operons that lack *cydX*, and CydZ encoded in more than 150 CydA_Qshort_ operons.

**Conclusions:**

This study provides a systematic analysis of bioinformatics techniques required for the unique challenges present in small protein identification and phylogenetic analyses. These results elucidate the prevalence of CydX throughout the Proteobacteria, provide insight into the selection pressure and sequence requirements for CydX function, and suggest a potential functional interaction between the small protein and the CydA Q-loop, an enigmatic domain of the cytochrome *bd* oxidase complex. Finally, these results identify other conserved small proteins encoded in cytochrome *bd* oxidase operons, suggesting that small protein subunits may be a more common component of these enzymes than previously thought.

**Electronic supplementary material:**

The online version of this article (doi:10.1186/1471-2164-15-946) contains supplementary material, which is available to authorized users.

## Background

Small protein research represents an emerging frontier in bioinformatics and proteomics. Relatively little attention has been paid to open reading frames coding for small proteins of fewer than 50 amino acids (aa), but in the last decade, small proteins have been discovered and characterized across a broad spectrum of life. Some examples include the 11–13 aa TAL proteins, which are required for embryonic development in Drosophila [1], the MntS, KdpF and AcrZ small proteins in *Escherichia coli* that play a role in intracellular manganese regulation [2], ion transport [3], and antibiotic resistance [4], respectively, and the SpoVM protein that recognizes cell curvature in *B. subtilis*[5]. Similarly, the short open reading frame (sORF) *ybgT* in the *E. coli* cytochrome *bd*-I oxidase operon encodes a 37 aa small protein labeled CydX that associates with the CydAB subunits of the complex and is required for oxidase activity [6, 7].

**Figure 1:**
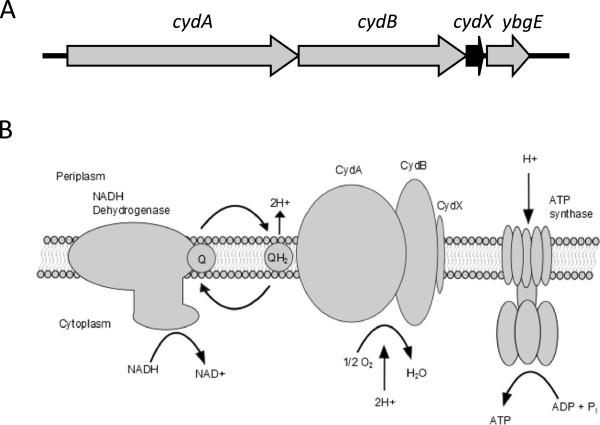
***cydABX*** **organization and function in** ***Escherichia coli*** **. (A)** Operon organization of the *cydABX* cytochrome *bd* oxidase operon. **(B)** Function of the CydABX complex in the electron transport chain.

Cytochrome oxidases are a broad family of integral membrane complexes that catalyze the terminal electron transfer in eubacterial and archaeal respiration [8]. These complexes couple the oxidation of either quinol or cytochrome *c* substrates with the reduction of O_2_ to water (Figure 1B). Cytochrome *bd*’s high O_2_ affinity allows it to efficiently scavenge oxygen to prevent damage to oxygen-sensitive enzymes, as well as permit growth in microaerobic and anaerobic environments. The complexes are also essential for survival under a number of stress conditions, including iron deficiency, nitrosative and peroxide stresses, exposure to cyanide, pressure, and high temperatures [9, 10]. Cytochrome *bd* oxidases support the Dsb pathway in catalyzing protein disulfide bonds for proper protein folding by re-oxidizing quinones reduced by DsbB in the activity of the pathway [11]. Furthermore, *bd* oxidases enhance the persistence of bacteria engulfed by macrophages [12], and are required for virulence of many bacterial pathogens [13, 14]. As such, cytochrome *bd* is under investigation as an antibiotic target [9].

Cytochrome *bd* oxidases have been studied for over 70 years; however the essential role of the CydX small protein in CydABX activity in *E. coli* and *B. abortus* was only recently discovered (Figure 1A, B) [6, 7, 15, 16]. This is due in part to the difficulties in identifying and characterizing small proteins using standard bioinformatics and biochemical techniques [17–19]. The common approach of matching an unidentified protein to a library of known proteins and domains is often inadequate to identify small proteins, which are largely unannotated, rarely characterized, and can be too small to contain common protein domains. Furthermore, it has been suggested that small proteins are frequently species-specific and may evolve independently when organisms are exposed to particular environmental conditions, obscuring typical phylogenetic examination with a combination of few protein sequences to analyze and an elevated variability in sequences [20]. Ultimately, this makes using typical bioinformatics approaches to identify and analyze small proteins error-prone and inadequate to resolve phylogenetic affinities.

Given that cytochrome *bd* oxidases are widely distributed and well conserved, we sought to determine the prevalence and phylogenetic relationships of CydX in species spanning the major Eubacterial divisions. Starting with the *E. coli* CydX sequence known to produce a functional protein, a survey for homologues was conducted using multiple homology-based bioinformatics tools. Upon completion, over 300 CydX homologues were identified, and sequence analysis of the small protein and of the larger subunits of the complex suggests an association between CydX and a region of the CydA protein called the Q-loop. Furthermore, two additional sORFs were identified that may code for distinct, analogous small Cyd proteins. Taken together, this study’s findings indicate that small transmembrane proteins are widespread members of the cytochrome *bd* oxidase complexes in Proteobacteria, that these proteins share a common functionality across divergent homologues, and that CydX and convergent small proteins may act in concert with the CydA Q-loop in the activity of the membrane-bound oxidoreductase.

## Results and discussion

### Identification of annotated and unannotated CydX homologues using tblastn and a hidden Markov model-based screen

**Figure 2:**
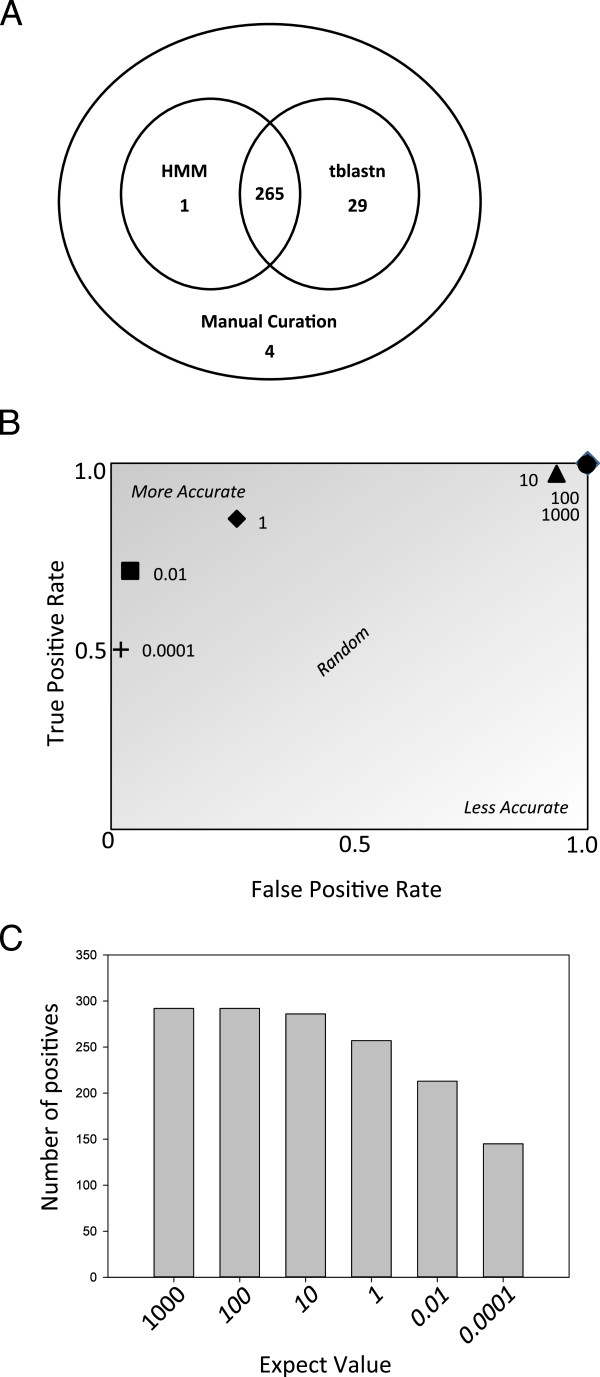
**Evaluating methods for accurately identifying CydX homologues in 1121 species of bacteria. (A)** Venn diagram of the number of CydX homologues identified by an HMM-based method (“HMM”), a tblastn screen of the NCBI microbial database using the CydX protein sequence as the query and an expect value of 1000 (“tblastn”), or by manual curation (“Missed”). **(B)** Receiver operating characteristic (ROC) plot of a tblastn screen of the microbial database using the CydX protein sequence as the query with different E-value cutoffs. **(C)** Graph of the number of CydX homologues identified in a tblastn screen of the microbial database using the CydX protein sequence as the query with different expect values. All tblastn searches were conducted using the NCBI BLAST Microbial Genomes site [45].

In order to investigate the extent of CydX conservation, complete genomes from 1095 taxa that span the major Eubacterial divisions were screened for potential homologues of the *E. coli* CydX protein. Two different bioinformatics techniques were used to identify homologues. The first technique was a series of searches for CydX homologues in each genome using the protein-nucleotide six-frame translation Basic Local Alignment Search Tool (tblastn) for microbial genomes, with the *E. coli* CydX protein sequence used as the query for each search. This method had the potential of identifying both annotated genes and unannotated ORFs that encode homologues. In order to maximize the probability of identifying divergent homologues, these tblastn searches were conducted using a very low stringency (E-value cutoff = 1000) with the low information filter turned off. On average, each search returned between 200–400 hits, with manual analysis of the tblastn results yielding between 1–10 likely candidates. For each candidate, the potential open reading frame was translated and screened for a significant Pfam hit for the ybgT_yccB small protein family. In a few cases, a potential homologue was identified in a search that did not give a significant Pfam hit, but showed substantial sequence similarity to CydX. In these instances, the distance of the ORF from the *cydAB* operon, the presence of an identifiable ribosome binding site, and the alignment of the small protein with the *E. coli* CydX sequence were used to determine if the ORF should be considered a homologue. In total, this method yielded 294 homologues (Figure 2A and Additional file 1).

To complement our tblastn analysis, we conducted a Hidden Markov Model (HMM)-based screen to identify potential homologues using the program HMMER [21]. This program was designed to search protein databases for homologues to a query sequence using a model of homology that typically outperforms blast search for homology specificity [22]. Following the methodology of the AMPHORA pipeline [23], we first built the HMM with a set of proteins that had been annotated as CydX or YbgT. From this profile alignment, we could query for additional sequences from the exomes of the 1095 genome sequences to detect homologues of CydX. Additionally, following the broad HMM screen, we tightened our search to the 4000 base pairs downstream of each *cydAB* operon to detect any previously unannotated ORFs through sixpack [24]. All ORFs identified in the region were then analyzed for the presence of a ybgT_yccB Pfam hit in the potential translation product. ORFs with a potential translation product containing a Pfam hit to the small protein family were considered a homologue. Using these screening methods, 266 CydX homologues were identified (Figure 2A and Additional file 1).

As expected, there was considerable overlap between the two methods. The tblastn screen yielded more positives, due to the fact that the HMM-based method missed homologues that are too divergent to give a significant Pfam result or are encoded outside of *cydAB* operons (Additional files 1 and 2a). However, the tblastn search also missed homologues (Figure 2A). In some cases, a tblastn screen using the *E. coli* CydX protein sequence as the query identified only one homologue in two closely related species. In these situations, a second tblastn search was conducted using the protein sequence of the homologue from the closely-related species as the query. In four species, this identified a new homologue (Table 1 and Additional file 3). Based on these results, it is possible that even more divergent CydX homologues may remain to be discovered.

**Table 1:** **Summary of CydX homologues identified in this study**

	Annotated	Unannotated	Total
**Tblastn + HMM**	221	74	295
**Manual curation**	0	4	4
**Leptospirillum**	2	0	2
**Plasmids**	3	2	5
**Total**	226	80	306

### Complementation of the Δ*cydX*mutant supports the accuracy of the homologue identification methods

**Figure 3:**
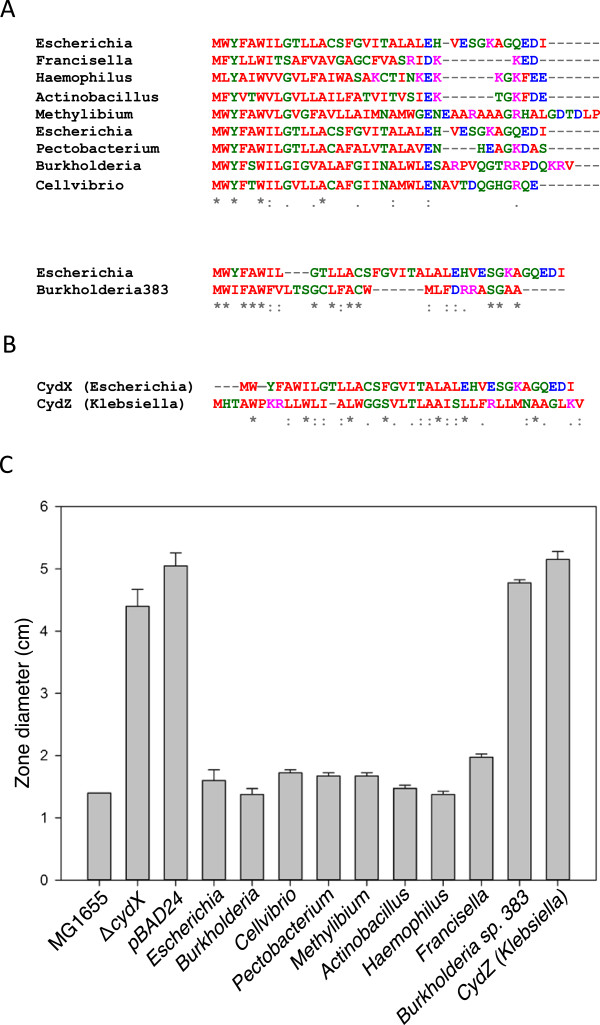
**Confirmation of functionality of CydX homologues. (A)** Alignment of protein sequences of CydX homologues from *Escherichia coli* and other bacteria species. The small protein from *Burkholderia sp.* 383 (“Burkholderia383”) is not thought to be a homologue and was included as a negative control for the assay. Based on its significant sequence divergence was included in a separate alignment. **(B)** Alignment of the *E. coli* CydX protein with the CydZ protein from *Klebsiella pneumoniae*. **(C)** Assay of complementation of the Δ*cydX* β-mercaptoethanol sensitivity phenotype by expression of potential CydX homologues, a false positive from the tblastn search (*Burkholderia sp. 383*), and an unrelated small protein (CydZ) from a different bacterial species. Sensitivity was measured using zones of inhibition, and the diameter of the zone after addition of 10 μL of 12 M β-mercaptoethanol to a plate of bacteria is shown. Species are as follows: *Escherichia coli* (“Escherichia”), *Pectobacterium atrosepticus* (“Pectobacterium”), *Burkholderia xenovorans* (“Burkholderia”), *Actinobacillus pleuropneumoniae* (“Actinobacillus”), *Burkholderia sp. 383* (“Burkholderia sp. 383”), *Klebsiella pneumoniae* (“Klebsiella”), *Cellvibrio japonicus* Ueda107 (“Cellvibrio”), *Methylibium petroleiphilum* PM1 (“Methylibium”), *Haemophilus influenzae* 10810 (“Haemophilus”), and *Francisella philomiragia* subsp. Philomiragia ATCC 25017 (“Francisella”). Alignments were generated using the program MUSCLE [57]. Amino acids are colored based on their properties at physiological conditions as follows: red amino acids are hydrophobic, green residues are hydrophilic, purple residues are positively-charged and blue residues are negatively-charged. ‘*’ indicates that the residues are identical in all sequences and ‘:’ and ‘.’, respectively, indicated conserved and semi-conserved substitutions as defined by MUSCLE.

To test the accuracy of our identification methods, we synthesized seven of the homologues identified in our screens and determined if they could functionally replace the CydX protein in *E. coli* by complementing the Δ*cydX* mutant. Four of these small proteins, identified in *Actinobacillus pleuropneumoniae*, *Burkholderia xenovorans*, *Methylibium petroleiphilum* PM1 and *Pectobacterium atrosepticus* were clear CydX homologues with significant Pfam hits (Figure 3A). One protein, encoded in *Francisella philomiragia* subsp*. philomiragia* ATCC25017, has a more divergent sequence but still returns a significant Pfam hit, while a sixth small protein, from *Haemophilus influenzae* 10810, has a divergent sequence and does not yield a Pfam hit (Figure 3A). The homologue from *Cellvibrio japonicas* Ueda107 was chosen as a representative of a few orphan homologues found to be encoded separately from a *cydAB* operon (Additional files 1 and 2a). We also tested a small protein identified by tblastn in *Burkholderia sp.* 383 that shows some sequence homology with CydX but lacks a significant Pfam hit and was ultimately scored as a negative, as well as an unrelated small Cyd protein identified in a *cydAB* operon in *Klebsiella pneumoniae* (Figure 3B). The ability of these small proteins to complement the *E. coli* Δ*cydX* mutant was assayed by transforming Δ*cydX* with a plasmid expressing each small protein, and testing the sensitivity of the transgenic strain to the reductant β-mercaptoethanol. Zone assays of these strains showed that all seven of the identified homologues complement the Δ*cydX* mutant, whereas the two negative control small proteins do not (Figure 3C). These results support the accuracy of our identification methods, provide evidence that the Pfam HMM for the CydX family is too stringent, and suggest that CydX homologues retain a similar functionality among divergent species.

### tblastn coupled with Pfam is an effective method for identifying small protein homologues

During the tblastn screen, we noticed that the statistics supporting many homologues that gave significant Pfam hits were much lower than those expected from tblastn searches using larger proteins. For example, the best hit in *Actinobacillus pleuropneumoniae* serovar 3 str. JL03, which was confirmed to be a true homologue based on complementation, yielded 27.7 bits with a blast expect value of 0.024. In comparison, a tblastn search using the *E. coli* CydA protein returned the corresponding *A. pleuorpneumoniae* CydA with 750 bits and an expect value of 0.0. The relatively low statistical support for many CydX homologues likely reflects the small size of the protein coupled with the sequence diversity observed between homologues. To evaluate the efficacy of our tblastn + Pfam-based screen, we conducted a receiver operating characteristic (ROC) analysis using tblastn data from all species surveyed [25]. The plot of the ROC showed that tblastn + Pfam is an effective method for identifying small protein homologues (Figure 2B), and that that E-value cutoffs between 0.01 and 1 are the most accurate setting for homologue identification. However, it is also clear that higher E-value cutoffs (100 or above) are required to identify divergent homologues, since ~10% of homologues are missed using a cutoff of 1 (Figure 2C).

### Sequence analysis of CydX homologues shows substantial diversity between proteins with few highly conserved residues

**Figure 4:**
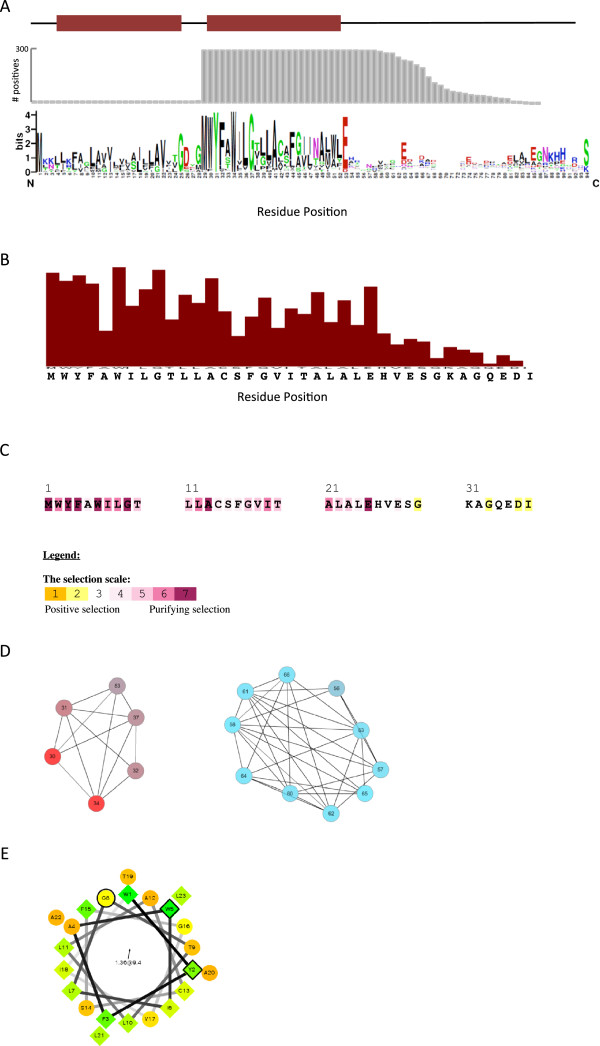
**Sequence analysis of the CydX protein family. (A)** Consensus sequence of CydX homologues compared to the presence of predicted transmembrane domains (red bars) and the number of homologues that contain amino acids at each position (grey bars). The sequence logo was created using a MUSCLE alignment [57] analyzed by the WebLogo program [57]. Amino acids are colored based on their properties at physiological conditions as follows: black amino acids are hydrophobic, green residues are hydrophilic, blue residues are positively-charged and red residues are negatively-charged. Transmembrane domains were predicted using the program TMHMM [56]. **(B)** Predicted evolutionary importance of each residue in CydX. Analysis performed using the Lichtarge Computational Biology Lab’s Universal Evolutionary Trace web server [57]. **(C)** Predicted selection pressure on each amino acid in the CydX protein. Analysis performed using the Selecton program. **(D)** Residues within the CydX protein that share mutual information. Analysis performed using the MISTIC program. Residues are colored based on conservation, with the amino acids in red positions in the alignment being conserved and blue amino acids showing less conservation. **(E)** Alpha-helical wheel project of the predicted transmembrane domain of the *E. coli* CydX protein [28]. The conserved residues Y3, W6 and G9 are outlined in black. The shapes the amino acids are based on their properties at physiological conditions as follows: hydrophobic residues are diamonds and hydrophilic residues are circles. The degree of hydrophobicity of diamond residues is also reflected in the color, with green being most hydrophobic and yellow being least hydrophobic, and a range of color between those depending on predicted hydrophobicity. Likewise, the degree of hydrophilicity of circle residues is reflected in the color, with red being most hydrophilic and light orange being least, and a range of color between those depending on predicted hydrophilicity.

The large number of CydX homologues identified in this study presented a unique opportunity to investigate amino acid sequence conservation in a widely-conserved small protein. A multiple sequence alignment (MSA) of 299 CydX homologues was used to create an amino acid sequence logo representing the relative conservation of residues in the protein (Figure 4A). The sequence logo shows that although much of the CydX protein is highly variable, there is a core region of higher homology containing a conserved tryptophan located at the N-terminal of a conserved hydrophobic α-helix (Figure 4A). Based on previous experimental results, the hydrophobic α-helix is thought to span the inner membrane [16, 26, 27], with the N-terminal and C-terminal of the protein on the cytosolic and periplasmic sides of the membrane, respectively [16]. For clarity, the *E. coli* sequence numbering will be used to demarcate the amino acids discussed here, with the N-terminal tryptophan being the sixth residue in the *E. coli* CydX protein. This conserved region contains other highly conserved residues including Y3 (all but one homologue), G9 (all but seven homologues) and E/D25 (either glutamate or aspartate in all but one homologue). Together, these residues make up the amino acid motif **Y**xx**W**xx**G**x_15_**E/D** that spans 97% of the homologues identified in this study. Of these four amino acids, Y3, W6 and G9 are predicted to be contained in the transmembrane α-helix (Figure 4A). An α-helical projection of the CydX hydrophobic region [28] shows that these residues may be localized to the same side of the helix (Figure 4E), suggesting that this may be the side of the α-helix that interacts with the other proteins in the CydABX complex. Outside of the core region, conservation significantly decreases, caused in part by the high variability in length of the C-terminal end of the small proteins (Figure 4A).

### CydX may contain distinct mini-domains

To further investigate the evolutionary significance of residues within the CydX protein, a CydX MSA was analyzed using two programs that consider both amino acid conservation and physio-chemical properties. One program, the Universal Evolutionary Trace, produced a real-valued trace in which the predicted evolutionary importance of each residue relative to the whole protein is calculated [29]. As expected, the results of this analysis coincided well with the amino acid conservation, with more highly conserved residues predicted to have greater evolutionary significance (Figure 4B). A second program, Selecton, was used to assess the ratio of nonsynonymous to synonymous mutations (K_a_/K_s_) and predict the type of selective pressure observed on each amino acid [30]. This analysis found that many residues in the N-terminal region of the protein are under purifying selection, whereas ratios near the C-terminal indicate that there is no selection, or possibly even positive selection, occurring (Figure 4C). Together, these results suggest that the CydX protein may have two mini-domains: the evolutionarily stable region containing the N-terminal amino acids plus the α-helix and the highly variable C-terminal region of the protein, which is relatively short in the *E. coli* CydX, but can reach up to 44 amino acids in some homologues. An examination of mutual information between residues in CydX using the Mutual Information Server to Infer Co-evolution (MISTIC) web server [31] found that, even at the lowest stringency, absolutely no mutual information was detected between the N-terminal residues and those in the C-terminus (Figure 4D). This result suggests that there is no co-evolution between these two regions of the protein, and is consistent with the idea that the CydX protein contains two protein mini-domains and that these regions may be functionally distinct.

### Mutational analysis confirms sequence plasticity of C-terminus

**Figure 5:**
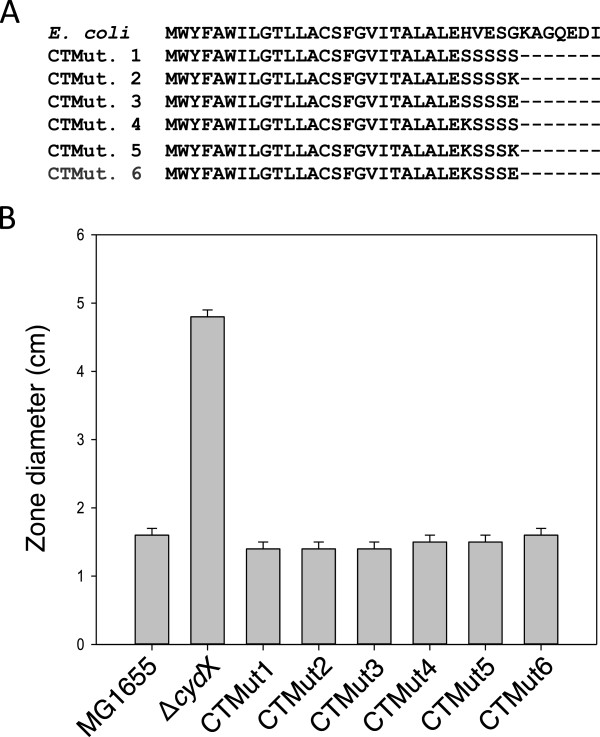
**Testing the functional importance of the CydX C-terminal amino acids. (A)** Alignment of the *E. coli* CydX protein sequence along with six mutant sequences containing mutated C-terminal amino acid sequences. **(B)** Assay of CydX function was conducted using a zone assay testing the sensitivity to β-mercaptoethanol. Sensitivity was measured using zones of inhibition, and the diameter of the zone after addition of 10 μL of 12 M β-mercaptoethanol to a plate of bacteria is shown. The average and standard deviation of zone sizes was calculated from at least three replicate plates. Alignments were generated using the program MUSCLE [57].

As an initial test of the functional importance of the CydX C-terminal region, a series of *E. coli* strains were constructed in which the amino acids following E25 were mutated in the *cydX* gene on the chromosome. The *cydX* genes in these strains were altered to encode either a six-serine tag at the C-terminus or a series of serines with flanking charged residues (Figure 5A). These residues were chosen in order to drastically alter the amino acid sequence while maintaining any general hydrophilic interactions within the periplasmic space and avoiding disruption of the orientation of the hydrophobic helix in the membrane. These strains were then tested for mutant phenotypes related to decreased CydX function, including mixed colony formation and sensitivity to β-mercaptoethanol. In all cases, the mutant CydX strains behaved like wild-type (unpublished data and Figure 5B), indicating that either there is no sequence specificity requirements for this region, the sequence requirements can be fulfilled by an essentially random series of hydrophilic amino acids, or this region of the protein is not required for CydX function under these conditions.

### CydX conservation suggests evolution of the small protein in the Proteobacteria

**Figure 6:**
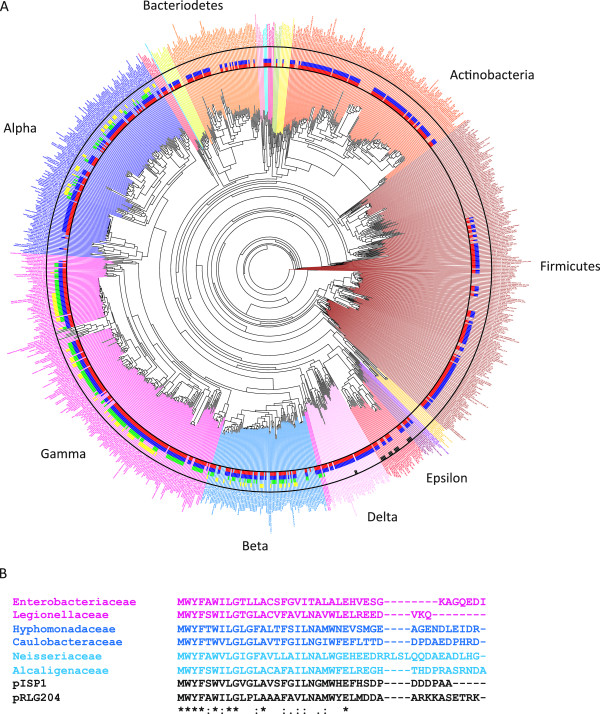
**Distribution of** ***cydA, cydB*** **,** ***cydX*** **and other** ***cyd*** **-related small proteins throughout bacteria. (A)** Phylogenetic tree of 1095 species from major Eubacterial clades overlaid with the presence of the different *cyd* genes in each species. Gene identification in a bacterial genome are labeled as follows: species adjacent to a red bar contain at least one *cydA* gene, to a blue bar contain at least one *cydB* gene, to a green bar contain at least one *cydX* gene, those adjacent to an yellow bar contain at least one *cydZ* gene, and those adjacent to a black bar contain at least one *cydY* gene. Major bacterial clades are labeled. The Alpha, Beta, Epsilon, Delta and Gamma labels identify the different classes in the Proteobacteria phylum. **(B)** Alignment of representative homologues identified from major bacterial clades. Gene names and sequences are shaded corresponding to the color used for that clade in the preceding phylogeny, while pISP1 and pRLG204 are not colored because they are not represented in the tree. Species are as follows: *Shigella flexneri* 2a str. 2457 T (“Enterobacteriaceae”), *Legionella pneumonophila* 2300/99 Alcoy (“Legionellaceae”), *Hyphomonas neptunium* ATCC15444 (“Hyphomonadaceae”), *Asticcacaulis excentricus* CB 48 (“Caulobacteraceae”), *Laribacter hongkongensis* HLHK9 (“Neisseriaceae”), *Archromobacter xylosoxidans* A8 (“Alcaligenaceae”), *Mariprofundus ferrooxydans* PV-1 1099921033905 (Mariprofundaceae), *Sphingomonas sp. MM-1* plasmid pISP1 (“pISP1”), and *Rhizonbium leguminosarum* bs. trifolii WSM2304 plasmid pRLG204 (“pRLG204”). Alignments were generated using the program MUSCLE [57]. ‘*’ indicates that the residues are identical in all sequences and ‘:’ and ‘.’, respectively, indicated conserved and semi-conserved substitutions as defined by MUSCLE.

Of the 1095 species screened in our original analysis, all 259 CydX-containing species were found to be members of the Alpha, Beta, and Gamma classes of the Proteobacteria. In contrast to the cladistically-limited distribution of CydX, CydA and CydB homologues were identified in species that range through almost all phyla included in the analysis (Figure 6A and Additional file 4). The difference in distribution between CydX and CydAB is consistent with the idea that CydAB evolved earlier than the CydX small protein. Given the evolutionary model that Alpha, Beta, and Gamma classes diverged after the earlier branching of Delta and Epsilonproteobacteria [32], the distribution of CydX suggests that it may have evolved in association with the *cydAB* operon in a progenitor of the Alpha, Beta, and Gammaproteobacteria clades.

Although no species outside of the Proteobacteria screened in our analysis contained an identifiable CydX homologue, a broader tblastn analysis was conducted in order to determine if this may be a false negative result due to the sampling of species chosen for this study. This search yielded only two species outside of Proteobacter, *Leptospirillum ferrooxidans* C2-3 and *Leptospirillum ferriphilum* ML-04, that contain an identifiable homologue (Table 1 and Additional file 5). These species are members of the Nitrospiraceae family in the phylum Nitrospirae. Given the phylogenetic distance between these *Leptospirillum* species and the other CydX-containing species we identified, of which all are contained in Proteobacter phylum, it is possible that these bacteria gained the *cydABX* operon through horizontal gene transfer.

### Phylogenetic analysis provides evidence of horizontal gene transfer

**Figure 7:**
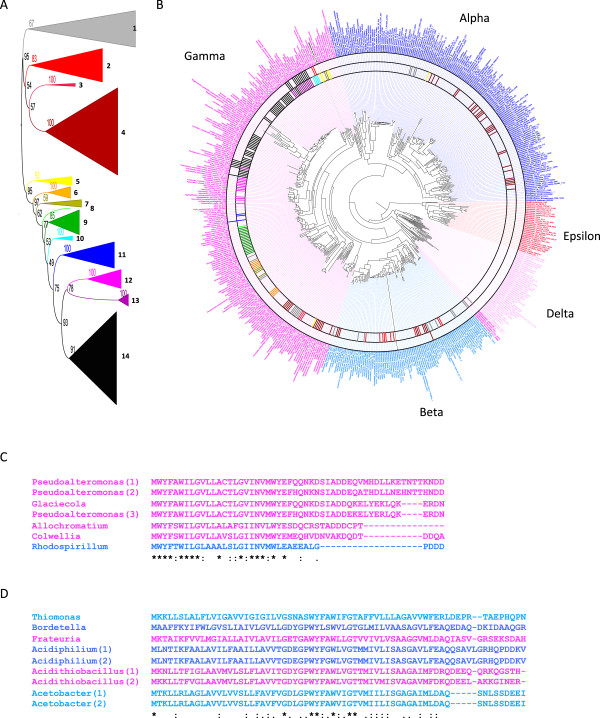
**Phylogenetic analysis of CydX. (A)** Phylogenetic analysis was conducted using concatenated CydABX protein sequences, and clades of CydABX sequences with strong statistical support are labeled by color. **(B)** Species containing specific CydABX sequences are labeled on the phylogenetic tree using bars of the same color as their clade in the phylogenetic analysis of the CydABX sequences. Species containing CydX homologues that are not contained in a *cydABX* operon are labeled with a black bar. The Alpha, Beta, Epsilon, Delta and Gamma labels identify the different classes in the Proteobacter phylum. **(C)** Alignment of protein sequences of CydX homologues grouped into the “yellow clade” in the phylogenetic analysis. **(D)** Alignment of select protein sequences of CydX homologues grouped into the “grey clade” in the phylogenetic analysis. Gene names and sequences are shaded corresponding to the color used for that clade in the preceding phylogeny. Species are as follows: *Pseudoalteromonas haloplanktis* TAC125 (“Psuedoalteromonas(1)”), *Pseudoalteromonas sp. SM9913* (“Pseudoalteromonas(2)”), *Glaciecola sp. 4H-3-7 + YE-5* (“Glaciecola”), *Pseudoalteromonas atlantica* T6c (”Pseudoalteromonas(3)”), *Allochromatium vinosum* DSM 180 (”Allochromatium”), *Colwellia psychrerythraea* 34H (“Colwellia”), *Rhodospirillum photometricum* DSM 122 (“Rhodospirillum”), *Thiomonas intermedia* K12 (“Thiomonas”), *Bordetella avium* 197 N (“Bordetella”), *Frateuria aurantia* DSM 6220 (“Frateuria”), *Acidiphillium cryptum* JF-5 (“Acidiphilium(1)”), *Acidiphillium multivorum* AIU301 (“Acidiphilium(2)”), *Acidithiobacillus ferrooxidans* ATCC 53993 (“Acidithiobacillus(1)”), *Acidithiobacillus caldus* SM-1 (“Acidithiobacillus(2)”), *Acetobacter pasteurianus* IFO 3283–01 (“Acetobacter(1)”), and *Acetobacter pasteurianus* IFO 3283-01-42C (“Acetobacter(2)”). Alignments were generated using the program MUSCLE [57]. ‘*’ indicates that the residues are identical in all sequences and ‘:’ and ‘.’, respectively, indicated conserved and semi-conserved substitutions as defined by MUSCLE.

To investigate the prevalence of CydX horizontal gene transfer, we attempted to create a phylogenetic tree based on CydX and superimpose this tree on a reference phylogenetic tree of the 1095 taxa screened in the study. In this way we could identify instances where the phylogenetic relationships between CydX homologues were incongruous with the overall phylogeny, which may be an indication of horizontal gene transfer. The maximum likelihood bootstrap values for the trees based solely on the CydX amino acid or DNA sequence were low and provided insufficient confidence to infer relationships among gene copies, let alone horizontal transfer events (unpublished data). These low bootstrap values are likely due to the limited sequence available for comparison and the high sequence variability between CydX homologues. To overcome this problem we took into consideration that, outside of a few orphan genes, there were no observed gains or losses of *cydX* independent of *cydAB*. Thus, the *cydABX* operon might be considered as one evolutionary unit, and a phylogeny could be constructed based on the concatenated sequences of all three proteins. A phylogenetic tree of 280 concatenated protein sequences was constructed using this methodology and produced a tree with 11 main clades with higher than 80% bootstrap support (Figure 7A). The CydX sequences within each operon were then aligned separately to identify sequence homologies that were unique for each clade. Protein alignments and sequence logos of the CydX proteins within the clades show that each contains shared and derived sequence motifs (synapomorphies) (Additional file 6), supporting the idea that the operon phylogeny accurately reflects that of CydX.

The CydX phylogeny was then mapped onto the bacterial phylogeny using a color-based scheme to designate clades (Figure 7B). This allowed for visual identification of instances of potential horizontal gene transfer of the *cydX* gene between species such that the interpretation of horizontal transfer implies fewer evolutionary events than hypotheses of ancestral evolution followed by an extensive history of gene loss and/or duplication under a parsimony framework [33]. Based on this analysis, there are at least two clades that show strong evidence for horizontal gene transfer of *cydX* (as *cydABX*). One example in which we have high confidence is the yellow clade of the *cydABX* phylogenetic tree. The high bootstrap values (93%) provide strong support of monophyly for this CydX lineage (Figure 7A). The species that contain yellow clade homologues, however, are widely divergent (Figure 7B), and although six of seven homologues are present in Gammaproteobacteria, one is found in Betaproteobacteria (Figure 7C). It seems most parsimonious, therefore, that this species, *Rhodospirillum photometricum* DSM 122, gained its *cydABX* operon through horizontal gene transfer. A second example is a group of N-terminal extension homologues that contain a second hydrophobic α-helix (Additional file 2B), which group together in the grey clade but are present in extremely diverse bacteria species in the Alpha, Beta and Gammaproteobacter (Figure 7B and D), suggesting horizontal gene transfer between species. Although our analysis yielded other examples of likely gene transfer, the evidence of a high rate of operon loss in multiple clades makes unequivocal distinctions difficult. Ultimately, however, it is likely that the *cydABX* operon has been transferred numerous times between closely related and divergent bacteria species.

One potential vehicle of horizontal gene transfer of CydX sequences is via plasmids. A tblastn analysis of plasmid sequences in the NCBI database yielded five plasmids that contain CydX homologues as well as the corresponding *cydA* and *cydB* genes (Table 1 and Additional files 1 and 5). In all but one case, the CydX homologue in each plasmid most closely aligns with the CydX homologue found in the known host bacteria species for that plasmid (unpublished data). In the case of the *Tistrella mobilis* KA081020-065 plasmid pTM1, however, we were unable to identify a potential *cydX* gene or *cydAB* operon in the bacterial genome. A multiple sequence alignment of the pTM1 CydX protein, however, showed that it has high sequence homology with the CydX protein found in *Rhodospirillum centenum* SW (Additional file 5B). One possible explanation of these results is that the *cydABX* operon from *R. centenum* SW was co-opted by the pTM1 plasmid, which then transferred bacterial host species to *T. mobilis*. Considering that both *T. mobilis* and *R. centenum* SW are members of the Rhodospirillaceae family, it is possible that a plasmid could have transferred between these closely related host species.

### The presence of *cydX*is coupled with the *cydA*_*Qlong*_allele

**Figure 8:**
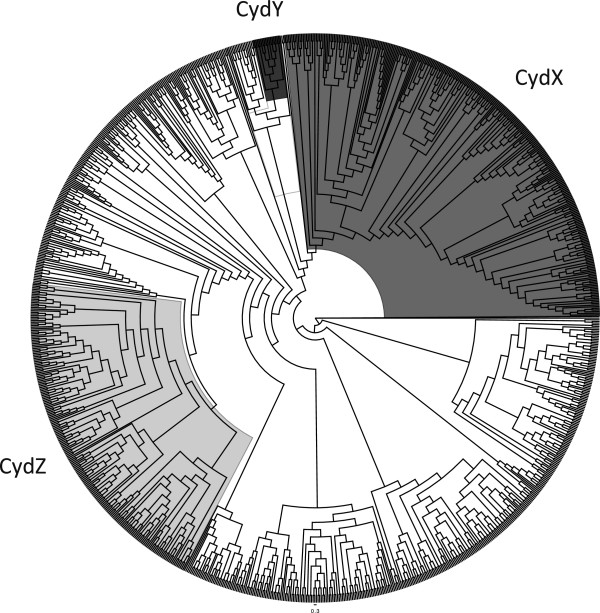
**Phylogenetic relationship between CydA protein sequence and presence of small Cyd proteins in the operon.** CydA DNA sequences were translated and aligned using MUSCLE, and the alignment was used to build a PHYLIP Neighbor Joining phylogenetic tree. Shading overlaying the phylogeny corresponds to CydA proteins that contain a *cydX*, *cydY*, or *cydZ* gene in the same operon.

**Figure 9:**
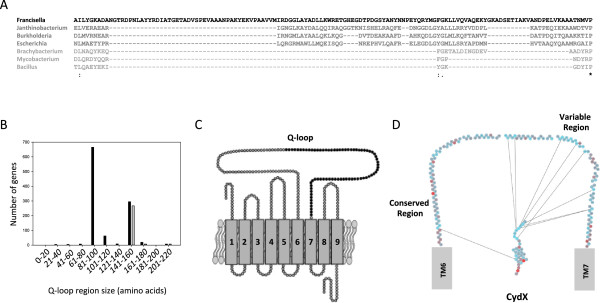
**Synteny between the** ***cydX*** **gene and the long Q-loop allele of** ***cydA*** **. (A)** Alignment of the Q-loop region from select CydA homologues. Sequences are shaded in a gradient going from longest Q-loop (darkest) to shortest Q-loop (lightest). **(B)** Histogram showing the number of CydA homologues containing Q-loops of increasing size (black bars) and the number of CydA proteins encoded in an operon also containing *cydX* (grey bars). **(C)** Diagram of the CydA protein containing the Q-loop. Residues shown in black are those that are present only in long Q-loop CydA variants. **(D)** Diagram showing mutual information shared between residues in the CydX protein, shown in its predicted orientation in the inner membrane, and the Q-loop, shown as the residues spanning transmembrane regions 6 (TM6) and 7 (TM7) of CydA. Lines between residues show high mutual information between residues. The conserved and variable regions of the Q-loop are labeled. Spaces between residues in the Q-loop region represent residues that are missing because they either show no mutual information or share mutual information with other Q-loop residues and not with CydX. A mutual information filter cutoff of 10 was used for this figure. Species are as follows: *Francisella philomiragia* subsp. philomiragia ATCC 25017 (“Francisella”), *Janthinobacterium sp. Marseille* (“Janthinobacterium”), *Burkholderia xenovorans* LB400 (“Burkholderia”), *Escherichia coli* 536 (“Escherichia”), *Brachybacterium faecium* DSM 4810 (“Brachybacterium”), *Mycobacterium marinum* M (“Mycobacterium”), and *Bacillus subtilis* subsp. spizizenii str. W23 (“Bacillus”). Mutual information was determined using the program MISTIC. Alignments were generated using the program MUSCLE [57]. ‘*’ indicates that the residues are identical in all sequences and ‘:’ and ‘.’, respectively, indicated conserved and semi-conserved substitutions as defined by MUSCLE.

As an initial investigation into the potential for co-variation between CydX and the other two Cyd proteins, a phylogeny of each of the larger proteins was constructed and then compared to the distribution of CydX. Although this analysis showed some correlation between CydB protein sequence and the presence of CydX, a very strong correlation was observed between CydX and CydA. When the presence of CydX is overlaid on a phylogeny created using the CydA sequence, there is a tight grouping of all CydA proteins encoded in operons that also encode CydX (Figure 8). This result strongly suggests that CydA proteins encoded in *cydABX* operons are different at the amino acid level from those encoded within operons lacking the *cydX* gene. Analysis of alignments of those CydA proteins having or lacking *cydX* identified a consistent sequence difference in a loop between transmembrane regions 6 and 7 in CydA called the Q-loop (Figure 9A, C) [9]. A plot of the length of the Q-loop region of CydA homologues versus the presence of *cydX* in the operon shows a separation of Q-loops into two major clades, with the shorter loops being primarily 81–100 amino acids, and a group of longer Q-loops ranging from 149–220 amino acids (Figure 9B). The length of the Q-loop shows a significant correlation with the presence of *cydX* in the *cydAB* operon. 89% of CydA_Qlong_ alleles reside in operons that contain *cydX*, and 99% of *cydX* homologues are encoded in an operon containing a CydA_Qlong_ allele. This close association between CydA_Qlong_ and CydX suggests that these regions may be functionally related.

### CydX and the variable domain of the Q-loop share mutual information

Although little is known about the function of the Q-loop in CydA activity, to further investigate the possibility that Q_long_ and CydX may have related functions, a mutual information analysis of CydX and the Q-loop region was conducted. If functional associations impose selective constraints on CydX and Q_long_ evolution, then these two regions would evolve in a correlated fashion and mutual information should be shared between these two protein regions. Consistent with this hypothesis, an analysis using MISTIC showed that there is substantial mutual information shared between residues in CydX and the Q_long_ region. The Q-loop is divided into two regions, with the N-terminal portion being more highly conserved while the C-terminus is highly variable [9]. Of these interactions, the highest mutual information was shared between residues in the C-terminal region of CydX and the variable region of Q_long_ (Figure 9D). Based on the current model of CydA and CydX orientation in the membrane [16, 34], both the CydX C-terminus and Q_long_ are located in the periplasmic space between the outer and inner membranes. Thus, a possible explanation for the high mutual information seen between these residues is that the C-terminus of the CydX protein may interact with the Q-loop, potentially stabilizing the interaction of CydX with the Cyd complex.

### Other conserved small proteins are encoded in *cydAB*operons

**Figure 10:**
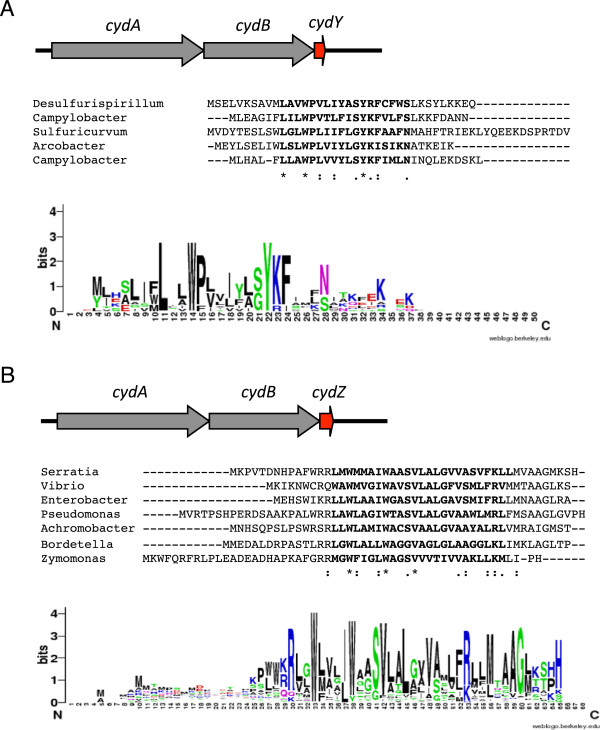
**New** ***cyd*** **-related small proteins identified in this study. (A)** The *CydY* small protein found in Epsilon and Deltaproteobacter species downstream of *cydAB* operons encoding CydA with a long Q-loop. **(B)** The CydZ small protein found in over 150 *cydAB* operons encoding CydA with a short Q-loop. Operon organization is shown on top of each figure, with an example alignment shown below followed by a consensus sequence logo shown at the bottom of the figure. Species are as follows: *Desulfurispirillum indicum* S5 (“Desulfurispirillum”), *Campylobacter concisus* 13826 (“Campylobacter”), *Sulfuricurvum kujiense* DSM 16994 (“Sulfuricurvum”), *Arcobacter butzleri* RM4018 (“Arcobacter”), *Campylobacter jejuni* subsp. doylei 269.97 (“Campylobacter”), *Serratia sp. AS12* (“Serratia”), *Vibrio parahaemolyticus* RIMD 2210663 (“Vibrio”), *Enterobacter aerogenes* KCTC 2190 (“Enterobacter”), *Pseudomonas aeruginosa* LESB58 (“Pseudomonas”), *Achromobacter xylosoxidans* A8 (“Achromobacter”), *Bordetella parapertussis* 12822 (“Bordetella”), *Zymomonas mobilis* subsp. mobilis ZM4 (“Zymomonas”). Sequence logos were generated using the program WebLogo [57]. Alignments were generated using the program MUSCLE [54]. ‘*’ indicates that the residues are identical in all sequences and ‘:’ and ‘.’, respectively, indicated conserved and semi-conserved substitutions as defined by MUSCLE.

Since 11% of the *cydAB* operons that contain CydA_Qlong_ do not encode CydX, we hypothesized that these operons may encode one or more previously uncharacterized small proteins that could potentially serve similar functions to CydX. To test this possibility, we manually screened the 35 CydA_Qlong_-containing operons to determine if there are other sORFs downstream of the *cydB* gene. In 15 species we identified a conserved sORF located downstream of *cydB* (Figure 6A, Additional files 1, 4 and 7) that could encode a small protein predicted to contain a transmembrane α-helix (Figure 10A and Additional file 7). Although the amino acid sequence of these small proteins is more divergent than CydX, all the proteins contain an absolutely conserved tryptophan located at the beginning of the conserved α-helix, similar to CydX (Figure 10A). All of the homologues we identified also contain strong ribosome binding sites (Additional file 7) and are encoded downstream of *cydB*, suggesting that they are transcribed with the operon and translated. In addition, when we examined the distribution of CydA proteins that contain this sORF, they grouped in a single clade adjacent to those containing CydX (Figure 8). Together, these data suggest that in these operons, a different small protein has evolved to function in the CydAB complex. We are referring to this protein as CydY.

The discovery of CydY raised the possibility that other *cydAB* operons, independent of the size of the CydA Q-loop, may also encode small proteins. A survey of sORFs downstream of *cydB* genes in all species yielded a large number of potential small proteins. Of these, one group of small proteins stood out due to its high degree of conservation. We identified this small protein in 162 *cydAB* operons that lack *cydX* (Figure 6A and Additional files 1, 4, and 8), and in many species it has already been identified. It is characterized by the Pfam family PF10617 (DUF2474), and we are referring to it as CydZ. Like CydX and CydY, CydZ contains a conserved hydrophobic α-helix as well as a conserved tryptophan at the beginning of the helix (Figure 10B). *cydZ* genes are also encoded downstream of *cydB* and are preceded by identifiable ribosome binding sites (Additional file 8), suggesting that the small protein is synthesized with the corresponding CydAB complex. Similarly, CydAB operons containing CydZ resolve together in the CydA phylogenetic tree (Figure 8), suggesting that there is a potential link between the sequence of these CydA proteins and the presence of CydZ. Consistent with a potential structural specificity between one type of CydA and a specific small protein, no operons were found to encode both CydZ and CydX even though the genomes of multiple taxa contain both *cydABZ* and *cydABX* operons. In many species the *cydZ* gene is located in operons known to encode cyanine-insensitive cytochrome *bd* oxidase complexes. The cyanide-insensitive oxidases (Cio) are a group of CydAB complexes that exhibit decreased sensitivity to hydrogen cyanide, and have been shown to lack the *d*-heme found in other cytochrome *bd* oxidases while containing a unique *b*-heme [35]. CydZ is encoded in the *cioAB* operons in *Zymomonas mobilis*, *Pseudomonas putida*, *Pseudomonas fluorescens* and the canonical *cio* operon in *Pseudomonas aeruginosa*. It will be interesting to determine if the CydZ protein plays a role in providing the unique biochemical characteristics of this class of complexes.

## Conclusions

### Identifying small protein homologues using bioinformatics

Of the two methods tested for the identification of small proteins, the tblastn analysis yielded a greater number of hits than the HMM method. One reason for the difference between the two methods is that the HMM screen examined only previously predicted ORFs and regions covering 4 kb downstream of *cydB*, meaning that it missed all “orphan” homologues. A second reason is that the HMM bioinformatics pipeline used a significant Pfam hit to the ybgT_yccB protein family as a screening step, meaning that any homologues that are too divergent for the ybgT_yccB Pfam reference sequence would be missed by this program. Although the HMM method was slightly less exhaustive, it was also much less time intensive than the tblastn analysis. It took an estimated 150–200 hours to complete and double-check the tblastn screen manually, compared to the ~10 hours it took to program and execute the HMM screen. In addition, this method should be applicable beyond studying well-conserved small proteins encoded within well-characterized operons. Minor modifications to the HMM method would allow for the screening for small protein homologues even if a Pfam designation has not been constructed for the protein in question, or if there is no known genomic region that would likely contain the small protein gene. In these cases, known small protein homologues could be used to create an HMM profile using hmmbuild [21], and this profile could be used to query a FASTA file containing regions of interest, such as the intergenic regions in a genome or a partitioned genome of interest, using hmmscan [21]. This process could also be used iteratively, with newly-identified homologues used to make more inclusive sequence profiles to use in future screens. Ultimately, although we found that the tblastn screen was more exhaustive in our study, it is clear that an automated survey such as the HMM method has advantages over a traditional tblastn screen.

Based on the ROC analysis of the tblastn screen, it seems that this method is an effective method for identifying small proteins. At lower E-value cutoffs however, an increasing number of true positives are missed, suggesting that although an E-value cutoff of 1 is the normal setting for tblastn, for small proteins the most useful value may be much larger. Ultimately, even at the highest E-value cutoff the tblastn analysis missed homologues (Figure 2A). In addition, our results suggest that the Pfam database and associated identification tools are not exhaustive for the CydX small protein. Similarly, a screen of CydX (YbgT/YccB family) homologues using the InterPro database [36], another commonly-used tool for protein identification, showed that 30 of the homologues identified in our study were not found in the database (data not shown), giving the database a success rate of ~90%. Altogether, these results suggest two things: one, a program with an iterative searching algorithm like PSI-BLAST for the translated nucleotide database would be a useful tool for identifying small proteins, and two, that manual curation remains an essential step in the accurate identification of small proteins and their homologues.

### Constructing a phylogeny for small proteins

The nucleotide and amino acid sequence limitations that cause small proteins to be challenging to identify using homology screens also lead to difficulties in construction of an evolutionary phylogeny. Although phylogenetic trees can be constructed, subsequent confidence metrics such as bootstrap values are low, suggesting that these trees may be unreliable predictions of gene relationships [37, 38]. When we tried to construct a tree using CydX protein sequences, subsequent consensus approaches inevitably yielded a “comb” tree in each attempt, indicating that there was little statistical support for the determinations of relatedness between each protein (unpublished data). We tried a number of methods to try to solve this problem, including limiting the analysis to the conserved core or variable C-terminal tail of amino acids for our analysis, and analyzing a set of artificial proteins constructed by concatenating the gene or amino acid sequences of each CydX homologue multiple times. Ultimately, however, none of these methods raised the bootstrapping values of our tree to reliable levels. The one method that did yield a reliable phylogenetic tree was the use of protein sequences consisting of a concatenation of CydA, CydB and CydX for each of the *cydABX* operons we identified. Not only did this method produce higher bootstrapping values, but analyses within well-supported clades suggest that the tree accurately identified clades of the CydX gene tree (Additional file 6). Thus, we found that information in contiguous regions that are inherited as a unit may provide information for phylogenetic resolution of gene trees when the genes themselves provide insufficient information to reconstruct their evolutionary histories. In the case of small proteins, inclusion of other genes encoded within the same operon may overcome the challenges of constructing a reliable phylogeny based solely on small protein sequence.

### CydX sequence evolution

The high sequence diversity observed between CydX homologues suggests some basic patterns in CydX evolution. First, essentially the entire CydX small protein exhibits sequence malleability. This is especially true for the highly-variable C-terminal portion of the protein, but the fact that only one residue in the entire protein, W6, was found to be absolutely conserved suggests that this is also true for the more conserved N-terminal region of the protein. Second, all homologues retain the core hydrophobic α-helix, suggesting that this secondary structure is critical for function. Taken together, these patterns suggest that the function of the CydX protein may be based on a few key residues, such as W6, Y3 and G9, whose conserved positions are required for participation in CydABX activity, and that the other residues in the protein exhibit sequence plasticity depending on their requirement for the positioning of those key amino acids. Thus, while amino acids in the helix are under selective pressure to remain hydrophobic in order to maintain the helix, the high variability observed for the C-terminal region could be the result of the fact that the only functional requirement for this region is to maintain loose interactions with the highly variable Q-loop. Further mutational analysis of CydX and the CydA Q-loop will be required to answer these questions.

### The evolution of the cytochrome *bd*oxidase small proteins

The distribution of species that contain CydA_Qlong_ compared to those that contain CydX suggest that CydA_Qlong_ may have evolved before the small protein. Species in Delta and Epsilonproteobacteria contain CydA_Qlong_, while lacking CydX. Considering that Alpha, Beta and Gammaproteobacteria are thought to be monophyletic and have diverged after Delta and Epsilon, it is possible that CydX evolved in a CydA_Qlong_ operon in the progenitor of the Alpha, Beta and Gamma clades. The presence of CydY in CydA_Qlong_ operons in the Delta and Epsilonproteobacteria also suggest that this small protein may have evolved independently in these classes after the divergence of the progenitor of Alpha, Beta and Gammaproteobacteria.

The discovery of sORFs encoding small proteins predicted to span the membrane in other *cydAB* operons raises the possibility that these proteins may be a more common component of CydAB complexes than previously thought. Although CydY and CydZ were the most conserved of these additional sORFs, our screen yielded a number of other hydrophobic small proteins potentially encoded adjacent to *cydAB* genes. For example, one intriguing hydrophobic small protein is encoded upstream of the *cydAB* genes in every *cydAB* operon that contains a CydA_Qlong_ allele but lacks CydX, including those that contain CydY. However, it is not clear from our analysis that this protein is expressed in the same operon as *cydAB*, leading us to not consider it a strong enough candidate to report here. It is clear, however, that small proteins may be a more common component of CydAB than previously thought, and that the high sequence variability observed for these proteins will require careful analysis to identify potential homologues in different operons.

## Methods

### Strain construction

All strains, plasmids and oligonucleotides used in this study are listed in Additional files 9, 10 and 11, respectively. All strains used were derivatives of the *E. coli* K-12 strain MG1655. The Δ*cydX*::kan strain was constructed as previously described [6]. To make the *cydX* C-terminus mutants, a *cydA*-*cydB*-*cydX*-Kan strain was first created by amplifying a kanamycin cassette from the plasmid pKD4, and transforming it into the recombinase-positive strain NM400 such that it would insert into the genome downstream of the *cydX* coding region. Genomic DNA from this strain was then used as template for the amplification of the kanamycin cassette with a mutagenic forward primer that mutated the 3′ end of the *cydX* gene. This PCR product was then transformed into NM400, and kanamycin-resistant colonies were screened for the mutated *cydX* allele using sequence-specific primers. All strains were confirmed by sequencing.

### Plasmid construction

To make the CydX homologue-overexpression plasmids, the short genes were created through overlapping PCR using primers that spanned one half of each gene plus 15 nucleotides for a region of primer overlap. The forward primer also contained an *Eco*RI restriction site at the 5′ end. These primers were used for template-less PCR, and the corresponding products were purified and digested with *Eco*RI. The digested PCR product was then ligated into pBAD24 plasmid that had been digested with *Eco*RI and *Sma*I, creating one *Eco*RI sticky end and a blunt end from the *Sma*I digestion. The ligation products were transformed into chemically-competent *E. coli* cells (Invitrogen), and ampicillin-resistant colonies were screened for the presence of insert by PCR. The sequence of all plasmids was confirmed by sequencing.

### Identification of annotated CydX homologues

Customized Perl scripts evoked the Web-based Eutils package [39] to download 1,095 genomes from Genbank. We downloaded the list of 1095 genomes used by Estes et al. [40] as we used their bacterial species phylogeny of these genomes for comparative analyses. Scripts using the BioPerl package [41] parsed the downloaded GenBank-formatted (gbk) sequence files and extracted each locus whose annotation feature tags ‘gene’ or ‘product’ matched (case-insensitive) ‘ybgt’. An amino acid alignment of the 38 annotated loci were made using MUSCLE [42]. We used this alignment to make a HMM profile using the HMMER 3.0 utility hmmbuild [21, 22] which provides a model of homology for the *cydX* locus. This model was then employed to query a FASTA file of all annotated amino acid sequences from the 1095 genomes for sequences with significant homology [22] to *cydX* that were not annotated with feature tags matching ‘ybgt’. We used an expectation value of 1e-3 to define our match cutoff, which yielded 198 matches. Each matched locus was subsequently compared to the Pfam-A database (downloaded March 5, 2013) [43] to check that it matched the family PF08173 (YbgT_YccB: Membrane bound YbgT-like protein).

### Identification of CydX homologues downstream of *cydAB*operons using an HMM-based analysis

The same pipeline (download genomes, parse genomes for loci annotated with features matching *cydA* or *cydB*, build hmm profile, use profile to query genomes) was employed to extract *cydA* and *cydB* sequences from the 1095 genomes. We scanned 50 base pairs (bp) upstream and 4000 bp downstream (4050 bp total) of the stop codon of each *cydB* locus for additional, possibly unannotated, loci using two open reading frame (ORF) detection programs: the EMBOSS 6.5.7.0 [24] utility sixpack and the Trinity (r2013-02-25) utility transcripts_to_best_scoring_ORFs.pl. The Trinity utility is typically for detection of likely coding regions in assembled contigs of RNA sequence reads, but we found it worked well to identify coding regions in DNA sequences that lack introns (such as bacterial DNA), as well. We scanned each detected ORF against the Pfam database to detect protein families in the ORFs, and we kept the ORF that was closest to the stop of the *cydB* locus having significant matches to Pfam families. We recognized the subset of these that matched the YbgT_YccB family PF08173 as previously unrecognized and unannotated *cydX* loci. We ran TMHMM 2.0 [44] on each ORF that was kept to detect likely trans-membrane domains.

### Identification of CydX homologues using tblastn

Homologues of the *E. coli* CydX protein were identified through tblastn searches of the genomes of bacteria species using the National Center for Biotechnology Information (NCBI) microbial database [45]. Only species that were labeled as “complete genomes” by NCBI were screened. Each species was searched individually, and except where noted, the *E. coli* CydX protein sequence was used as the query sequence. The low complexity regions filter was turned off in all cases. An E-value cutoff (Expect value) of 1000 was used in all cases except for searches used in the receiver operating characteristic (ROC) analysis. For the ROC analyses, individual tblastn searches were conducted for each species using a series of Expect values: 1000, 100, 10, 1, 0.01 and 0.0001. Positive hits from the tblastn search were screened for the presence of a significant match to the ybgT_yccB Pfam family using the EMBL-EBI Pfam server [46].

### Manual curation

Unannotated potential homologues were identified through the NCBI Gene database [47]. CydB protein YP numbers were used to direct the search, and the downstream nucleotide sequence up to ~500 nt was downloaded and used as input in ORF Finder [48] using the bacterial genetic code. Open reading frames were screened using their predicted protein sequences run through TMHMM v2.0 to verify the presence of the conserved α-helix and through Pfam Sequence Search [49] and tblastn to determine if the ORF contained a predicted protein with high similarity to previously identified likely homologues.

### Phylogenetic analysis of CydX homologues

Bayesian analyses of phylogeny were conducted to estimate relationships among gene copies of the *cydA*, *cydB*, and *cydX* loci. Copies of each locus were extracted from the genomes using the methods above were aligned using the HMMER program hmmalign. Amino acid alignments were reverse-translated to DNA alignments for phylogenetic analyses using custom Perl scripts. Models of sequence evolution for the loci individually and for the loci concatenated into the *cydABX* operon were selected using MrModelTest [50] which compares 24 different models of nucleotide evolution and selects the model that minimizes the Akaike Information Criterion (AIC) score [51]. The Bayesian analysis of phylogeny was carried out using MrBayes v3.2.1 × 64 [52]. We ran separate analyses on each locus and on the concatenated alignment. For each analysis, we ran 50,000,000 iterations of four chains of the Markov Chain Monte Carlo (MCMC) to reach stationarity, from which we sampled every 1000 iterations to reduce temporal autocorrelations among samples from the joint posterior distribution of gene tree topologies, branch lengths, and nucleotide model parameters. Based on this sample from the posterior distribution, we inferred the MrBayes ‘allcompat’ consensus tree.

For each consensus tree (one each for the *cydA*, *cydB*, *cydX*, and concatenated operon analyses), we classified each gene copy into one of the five largest clades, as characterized by the R script. We then mapped each operon, color coded by clade membership, onto the bacterial species tree of 1095 genomes provided by Estes et al. [40]. Estes et al.’s phylogenomic analysis was a likelihood-based supermatrix analysis of 264 protein-coding loci selected from an MCL analysis of orthology [53]. Based on the operon clade membership, we could thus discern the evolutionary history of each operon copy in relation to the species-level genome phylogeny using a parsimony framework that selected the operon evolutionary history that minimized the number of evolutionary events (operon duplication, loss, or horizontal transfer).

### CydA phylogeny

Using the UGENE [54] v1.13 software toolkit [54], all CydA DNA sequences were aligned using MUSCLE, with the setting “Translate to amino when aligning” ticked and using the Bacterial and Plant Plastid Code translation table, with all other settings default. The resulting alignment was used to construct a PHYLIP Neighbor Joining phylogenetic tree using the F84 distance matrix and other settings default.

### Sequence analysis of CydX homologues

Helix prediction was through TMHMM v2.0 [55] and Jpred 3 [56] web servers. TMHMM predictions were conducted using individual CydX amino acid sequences with standard settings, and Jpred predictions were based upon a MUSCLE MSA [42] of 300 CydX homologues entered into the Jpred web server, with both tools using default settings.

Visualization of CydX protein sequences was achieved using the Berkeley WebLogo web server [57] and input of CydX amino acid sequences aligned using MUSCLE. For some figures, manual curation of the MSA was used to remove divergent sequences, such as those with N-terminal extensions, as they were too scarce to provide useful sequence data. Furthermore, some primarily gapped positions caused by sequences with insertions were removed from the alignment to provide a cleaner view.

An evolutionary trace was conducted through Lichtarge Computational Biology Lab’s Universal Evolutionary Trace and web server [29]. As no structural data exist for CydX and the larger cytochrome *bd* complex, real-valued evolutionary traces using the *E. coli* CydX protein sequence with blastp, and a CydX MUSCLE MSA without blastp were both run with the *E. coli* CydX sequence set as the reference sequence in both cases. It was seen again that blastp missed many unannotated homologues, and setting less stringent E-values to include more divergent homologues also began incorporating non-CydX sequences.

To evaluate selection pressure on specific residues, the Selecton web server [30] was used to calculate K_a_/K_s_ ratios and test their significance. Lacking a repeatable phylogenetic tree for all CydX, 53 sequences encompassing multiple representatives from each major clade of CydX in the CydABX phylogenetic tree were selected, and their DNA sequences were used as input. The program was executed using the MEC model with the JTT substitution matrix, and the program was run at “high precision” settings. Upon completion, the program was run again with a null model to verify statistical significance.

Potential co-evolution between residues in CydX and between CydX and CydA was investigated using the MISTIC web server [31]. To use this program, a multiple sequence alignment was first constructed of the sequences in question using the MUSCLE server [42].

### Zone assays

Zone assays were conducted to test strains’ sensitivity to β-mercaptoethanol (Sigma-Aldrich). 200 μL of overnight cultures were diluted into 3 mL of top agar at 55°C. Strains that contained plasmid were plated on LB Amp plus 0.2% arabinose, while those that lacked plasmid were plated on LB plus 0.2% arabinose. Sterilized filter paper disks were placed in the center of the plate, and a 10 μL aliquot of β-mercaptoethanol was added to each disk. Plates were then grown overnight at 30°C under aerobic conditions before scoring zone diameters.

### Availability of supporting data

The data sets supporting the results of this article are contained within the additional files included with this article.

## Authors’ information

David J Hearn and Matthew R Hemm are co-senior authors.

## Electronic supplementary material

Additional file 1: **Lists the orientation, coordinates, DNA and protein sequences for all CydA, CydB, CydX, CydY and CydZ homologues identified in this study.** (XLSX 2 MB)

Additional file 2: **“Orphan” and N-terminal extension homologues.** (A) Genomic organization and protein sequence alignment of two homologues identified that are not located within *cydABX* operons. (B) Genomic organization and protein alignment of a representative of a group of CydX homologues identified in this study that contain two predicted transmembrane domains. The residues in each transmembrane domain are bolded. Species are as follows: *Acidithiobacillus ferrooxidans* ATCC 53993 (“Acidithiobacillus”), *Burkholderia phytofirmans* PsJN (“Burkholderia”), *Cellvibrio japonicus* Ueda107 (“Cellvibrio”), and *Escherichia coli* (“Escherichia”). Alignments were generated using the program MUSCLE [57]. ‘*’ indicates that the residues are identical in all sequences and ‘:’ and ‘.’, respectively, indicated conserved and semi-conserved substitutions as defined by MUSCLE. (PDF 64 KB)

Additional file 3: **Homologues missed by the tblastn + Pfam screen.** Protein sequence alignments of two homologues missed by the tblastn screen (shaded in grey) compared to their closest homologue (shaded in black) identified by the screen and the *E. coli* CydX sequence (shaded in black) used as the query sequence in the analysis. Species are as follows: *Escherichia coli* (“Escherichia”), *Haemophilus ducreyi* 35000HP (“H. ducreyi”), *Hamophilus influenzae* 86-028NP (“H. influenzae”), *Francisella cf. novicida* Fx1 (“F. cf. novicida”), and *Francisella philomiragia* subsp. philomiragia ATCC 25017 (“F. philomiragia”). Alignments were generated using the program MUSCLE [54]. ‘*’ indicates that the residues are identical in all sequences and ‘:’ and ‘.’, respectively, indicated conserved and semi-conserved substitutions as defined by MUSCLE. (PDF 29 KB)

Additional file 4: **Phylogenetic distribution of Cyd genes.** Species containing specific *cydA*, *cydA*_Qlong_, *cydB*, *cydX*, *cydY* and *cydZ* sequences are labeled on the phylogenetic tree using bars of the designated color. If a species contains more than one *cyd* operon, the operons are separated on parallel rings aligned with the species name. (PDF 273 KB)

Additional file 5: **Alignments of homologues potentially related through horizontal gene transfer.** (A) CydX homologues with high sequence homology found between the divergent species *Leptospirillum ferrooxidans* C2-3 (“Leptospirillium”) and *Sideroxydans lithotrophicus* ES-1 (“Sideroxydans”). (B) CydX homologues with high sequence homology found in *Rhodospirillum centenum* SW (“Rhodospirillum”) and the *Tistrella mobilis* KA081020-065 plasmid pTM1 (“pTM1”). Alignments were generated using the program MUSCLE [54]. ‘*’ indicates that the residues are identical in all sequences and ‘:’ and ‘.’, respectively, indicated conserved and semi-conserved substitutions as defined by MUSCLE. (PDF 36 KB)

Additional file 6: **Sequence logos and alignments of CydX homologues found in each clade identified by the phylogenetic analysis.** Each clade is identified by the color and number. A sequence logo is shown for the clade when the number of proteins in the clade were sufficient for logo determination. An alignment of the homologues is shown at the bottom. Sequence logos were generated using the program WebLogo [55]. Alignments were generated using the program MUSCLE [54]. ‘*’ indicates that the residues are identical in all sequences and ‘:’ and ‘.’, respectively, indicated conserved and semi-conserved substitutions as defined by MUSCLE. (PDF 331 KB)

Additional file 7: **Lists the orientation, coordinates, gene spacing and predicted ribosome binding sites of CydY homologues identified in this study.** (XLSX 57 KB)

Additional file 8: **Lists the orientation, coordinates, gene spacing and predicted ribosome binding sites of CydZ homologues identified in this study.** (XLSX 90 KB)

Additional file 9: **Lists the bacterial strains used in this study.** (XLSX 16 KB)

Additional file 10: **Lists the plasmids used in this study.** (XLSX 25 KB)

Additional file 11: **Lists the oligomers used in this study.** (XLSX 31 KB)

## Abbreviations

aa: Amino acid
ORF: Open reading frame
sORF: Short open reading frame
tblastn: Protein-nucleotide six-gram translation Basic Local Alignment Search Tool
ROC: Receiver operating characteristic
MISTIC: Mutual Information Server to Infer Co-evolution
Q_long_: The CydA long Q-loop
CydA_Qlong_: CydA protein containing the long Q-loop
Q_short_: The CydA short Q-loop.
