# Supplementary material for: Conservation analysis of the CydX protein yields insights into small protein identification and evolution
Source: BMC Genomics. 2014 Dec 5;15(1):946. doi: 10.1186/1471-2164-15-946 (PMC4325964; doi:10.1186/1471-2164-15-946)
Supplement: Supplementary file 2 — Additional file 2: “Orphan” and N-terminal extension homologues. (A) Genomic organization and protein sequence alignment of two homologues identified that are not located within cydABX operons. (B) Genomic organization and protein alignment of a representative of a group of CydX homologues identified in this study that contain two predicted transmembrane domains. The residues in each transmembrane domain are bolded. Species are as follows: Acidithiobacillus ferrooxidans ATCC 53993 (“Acidithiobacillus”), Burkholderia phytofirmans PsJN (“Burkholderia”), Cellvibrio japonicus Ueda107 (“Cellvibrio”), and Escherichia coli (“Escherichia”). Alignments were generated using the program MUSCLE [57]. ‘*’ indicates that the residues are identical in all sequences and ‘:’ and ‘.’, respectively, indicated conserved and semi-conserved substitutions as defined by MUSCLE. (PDF 64 KB) [file 12864_2014_6987_MOESM2_ESM.pdf]

A

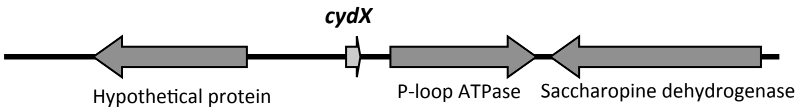


```
Escherichia       MWYFAWILGTLLACSFGVITALALE----------HVESGKAGQEDI--
Burkholderia      MWYFTWILGIGVALGFGIINVMWLEAGDKFTRDPQRARAVPATPEDAPS
                  ****:****  :* .**:*..: **          .. :  *  **
```

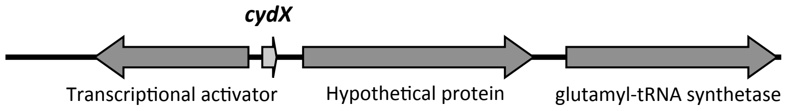


```
Escherichia       MWYFAWILGTLLACSFGVITALALEHV--ESGKAGQEDI
Cellvibrio        MWYFTWILGVLLACAFGIINAMWLENAVTDQGHGRQE--
                  ****:****.****:**:*.*: **:.  :.*:. **
```

B

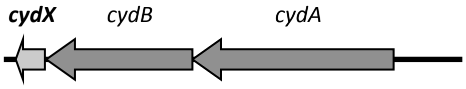


```
Escherichia         ----------------------------MWYFAWILGTLLACSFGVITALALEHVESGKAGQEDI--
Acidithiobacillus   MKNLLTFIGLAAVMVLSLFLAVITGDYGPWYFAWLVGTTMIVLISAAGAIMFDRQDEEQQRKQGSTH
                                                 *****::** :   :..  *: ::. :. :  ::.
```

Additional File 1
