# Supplementary material for: Conservation analysis of the CydX protein yields insights into small protein identification and evolution
Source: BMC Genomics. 2014 Dec 5;15(1):946. doi: 10.1186/1471-2164-15-946 (PMC4325964; doi:10.1186/1471-2164-15-946)
Supplement: Supplementary file 3 — Additional file 3: Homologues missed by the tblastn + Pfam screen. Protein sequence alignments of two homologues missed by the tblastn screen (shaded in grey) compared to their closest homologue (shaded in black) identified by the screen and the E. coli CydX sequence (shaded in black) used as the query sequence in the analysis. Species are as follows: Escherichia coli (“Escherichia”), Haemophilus ducreyi 35000HP (“H. ducreyi”), Hamophilus influenzae 86-028NP (“H. influenzae”), Francisella cf. novicida Fx1 (“F. cf. novicida”), and Francisella philomiragia subsp. philomiragia ATCC 25017 (“F. philomiragia”). Alignments were generated using the program MUSCLE [54]. ‘*’ indicates that the residues are identical in all sequences and ‘:’ and ‘.’, respectively, indicated conserved and semi-conserved substitutions as defined by MUSCLE. (PDF 29 KB) [file 12864_2014_6987_MOESM3_ESM.pdf]

```
Escherichia        MWYFAWILGTLLACSFGVITALALEHVESGKAGQEDI
H. ducreyi         MFYVTWVLGILLAILFATVVTIGIE-----KSGKFDE
H. influenzae      MLYAIWVVGVLFAIWASAKCTINKE-----KKGKFEE
                   * *  *::* *:*   ..  ::  *     * *: :
```

```
Escherichia        MWYFAWILGTLLACSFGVITALALEHVESGKAGQEDI
F. cf. novicida    MYYLAWIISAGLAVTVGCFVATRLEKKEDNSK-----
F. philomiragia    MFYLLWITSAFVAVGAGCFVASRIDKKED--------
                   *:*: ** .: :*   * :.*  ::: *.
```

Additional File 2
