# Supplementary figures and images for: Conservation analysis of the CydX protein yields insights into small protein identification and evolution
Source: BMC Genomics. 2014 Dec 5;15(1):946. doi: 10.1186/1471-2164-15-946 (PMC4325964; doi:10.1186/1471-2164-15-946)

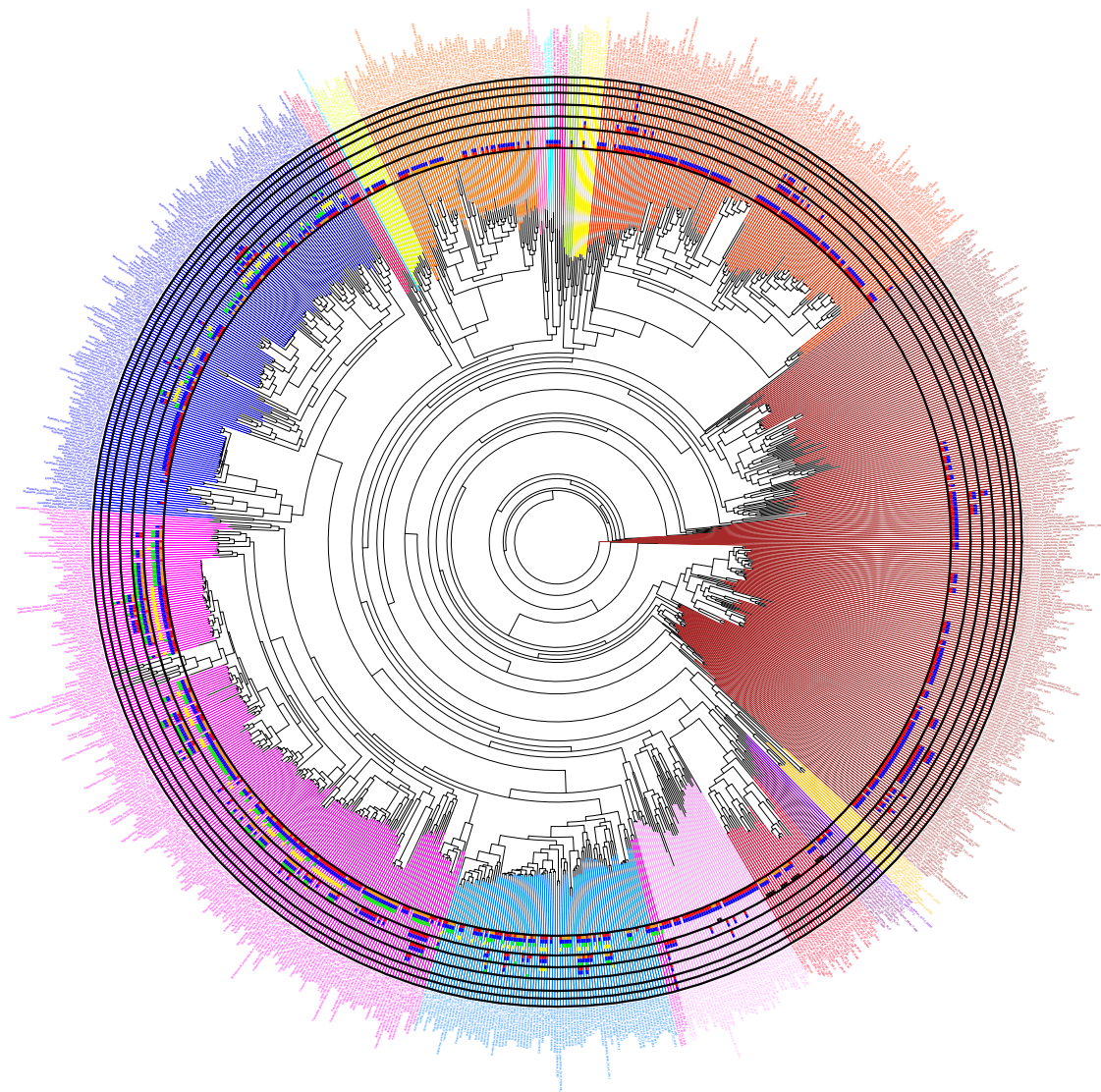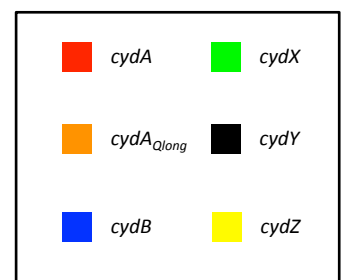

Supplement: Supplementary file 4 — Additional file 4: Phylogenetic distribution of Cyd genes. Species containing specific cydA, cydA Qlong, cydB, cydX, cydY and cydZ sequences are labeled on the phylogenetic tree using bars of the designated color. If a species contains more than one cyd operon, the operons are separated on parallel rings aligned with the species name. (PDF 273 KB) [file 12864_2014_6987_MOESM4_ESM.pdf]
