# Supplementary material for: Conservation analysis of the CydX protein yields insights into small protein identification and evolution
Source: BMC Genomics. 2014 Dec 5;15(1):946. doi: 10.1186/1471-2164-15-946 (PMC4325964; doi:10.1186/1471-2164-15-946)
Supplement: Supplementary file 5 — Additional file 5: Alignments of homologues potentially related through horizontal gene transfer. (A) CydX homologues with high sequence homology found between the divergent species Leptospirillum ferrooxidans C2-3 (“Leptospirillium”) and Sideroxydans lithotrophicus ES-1 (“Sideroxydans”). (B) CydX homologues with high sequence homology found in Rhodospirillum centenum SW (“Rhodospirillum”) and the Tistrella mobilis KA081020-065 plasmid pTM1 (“pTM1”). Alignments were generated using the program MUSCLE [54]. ‘*’ indicates that the residues are identical in all sequences and ‘:’ and ‘.’, respectively, indicated conserved and semi-conserved substitutions as defined by MUSCLE. (PDF 36 KB) [file 12864_2014_6987_MOESM5_ESM.pdf]

A

```
Leptospirillium       MWYFVWIVGVTMAVTLSVMHALWFEIQEDQVTIDRLRVDGKY
Sideroxydans          MWYFAWILGVSMAILLAIVNTMMCDAQACVTKEESSDKE
                      ****.**:**:**: *::::::  : *   :  :
```

B

```
pTM1                  MWYFAWLLGLPLACAFAVLNAMWFEMKEDAAEGQP-----
Rhodospirillium       MWYFAWLLGLPLAVVFAVLNGLWFELREDAARGQPDPDAF
                      ************* .*****.:***:.**** ***
```

Additional File 3
