# Supplementary material for: Conservation analysis of the CydX protein yields insights into small protein identification and evolution
Source: BMC Genomics. 2014 Dec 5;15(1):946. doi: 10.1186/1471-2164-15-946 (PMC4325964; doi:10.1186/1471-2164-15-946)
Supplement: Supplementary file 6 — Additional file 6: Sequence logos and alignments of CydX homologues found in each clade identified by the phylogenetic analysis. Each clade is identified by the color and number. A sequence logo is shown for the clade when the number of proteins in the clade were sufficient for logo determination. An alignment of the homologues is shown at the bottom. Sequence logos were generated using the program WebLogo [55]. Alignments were generated using the program MUSCLE [54]. ‘*’ indicates that the residues are identical in all sequences and ‘:’ and ‘.’, respectively, indicated conserved and semi-conserved substitutions as defined by MUSCLE. (PDF 331 KB) [file 12864_2014_6987_MOESM6_ESM.pdf]

Clade 1 (Grey clade)

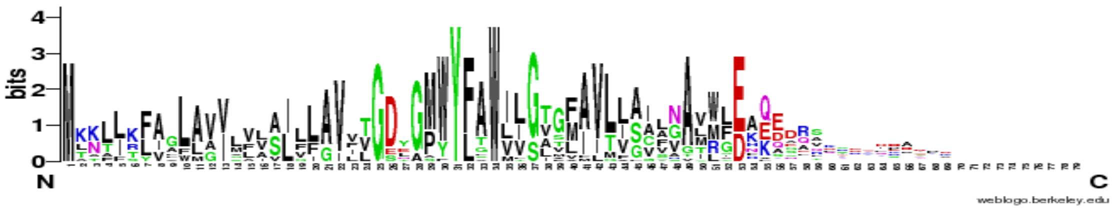


```
NC_009749-YP_001427637-2-YP_00      ----------------------------MYYLAWIISAGLAVTVGCFVATRLEKKEDQSK-------------------
NC_007880-YP_512984-YP_512983-      ----------------------------MYYLAWIISAGLAVTVGCFVATRLEKKEDQSK-------------------
NC_008601-YP_897857-YP_897856-      ----------------------------MYYLAWIISAGLAVTVGCFVATRLEKKEDQSK-------------------
NC_017450-YP_005825098-YP_0058      ----------------------------MYYLAWIISAGLAVTVGCFVATRLEKKEDNSK-------------------
NC_015696-YP_004646713-YP_0046      ----------------------------MFYLLWITSAFVAVGAGCFVASRIDKKED----------------------
NC_017094-YP_005468840-YP_0054      ----------------------------MWYFVWIVGVTMAVTLSVMHALWFEIQEDQVTIDRLRVDGKY---------
NC_016616-YP_005028130-YP_0050      ----------------------------MWYFTWILGLGFAVLLAVVNALWLEAKHTQE--------------------
NC_014147-YP_003626717-YP_0036      ----------------------------MWYFAWILGLGFAVLLAVVSAVWVEFEDSRRDGFNHPDDDQK---------
NC_009524-YP_001279232-YP_0012      ----------------------------MWYFAWILGLGFAVLLAIVNAVWLEHEQGRLDSYKPKPKPSENPNLNKTLK
NC_009446-YP_001209030-YP_0012      ----------------------------MWYLTWLVGLGFAVLFAILVALWGEFAQKRTENFAPKLPEKEDAV------
NC_012559-YP_002796245-YP_0027      ----------------------------MWYFAWVLGIGFAVLLAILNALWGEHEEDRRLSLQQDAEADLHG-------
NC_008702-YP_932736-YP_932735-      ----------------------------MWYFTWVLGIGFAVLLAILNALWGEHEDARAQPAADDAAPDLAPDAARP--
NC_007908-YP_523610-YP_523611-      ----------------------------MWYFAWILGVSFAVLLAILNAMWGENEEARAQARSPDA-------------
NC_016830-YP_005208492-YP_0052      ----------------------------MWYFAWMLGVGFALLLAILNAMWGENEAGRAMNDRDEGQP-----------
NC_015379-YP_004353799-YP_0043      ----------------------------MWYFAWMLGVGFALLLAILNAMWGENEAGRAMSDRDEAQP-----------
NC_014394-YP_003848159-YP_0038      ----------------------------MWYFAWVLGVSAAVMFAVLNAMWYEAQECAQIRGHDRAGLHSLR-------
NC_013959-YP_003523776-YP_0035      ----------------------------MWYFAWILGVSMAILLAIVNTMMCDAQACVTKEESSDKE------------
NC_014153-YP_003641759              MKKLLSLALFLVIGAVVIGIGILVGSNASWYFAWIFGTAFFVLLLAGAVVWFERLDEPRTAEPHQPN------------
NC_011206-YP_002219519-YP_0022      MKNLLTFIGLAAVMVLSLFLAVITGDYGPWYFAWLVGTTMIVLISAAGAIMFDRQDEEQQRKQGSTH------------
NC_015850-YP_004749631-YP_0047      MKKLLTFVGLAAVMVLSLFLAVVTGDYGPWYFAWLVGTTMIVMISVAGAVMFDKQDEELAKKGINER------------
NC_017150-YP_005501971-YP_0055      MTKLLRLAGLAVVLVVSLLFAVFVGDLGPWYFAWVIGTVMIILISGAGAIMLDAQSNLSSDEEI---------------
NC_013209-YP_003188057-YP_0031      MTKLLRLAGLAVVLVVSLLFAVFVGDLGPWYFAWVIGTVMIILISGAGAIMLDAQSNLSSDEEI---------------
NC_009484-YP_001234760-YP_0012      MLNTIKFAALAVILFAAILLAVVTGDEGPWYFGWLVGTMMIVLISASGAVLFEAQQSAVLGRHQPDDKV----------
NC_015186-YP_004283908-YP_0042      MLNTIKFAALAVILFAAILLAVVTGDEGPWYFGWLVGTMMIVLISASGAVLFEAQQSAVLGRHQPDDKV----------
NC_017033-YP_005377664-YP_0053      MKTAIKFVVLMGIALLAIVLAVILGETGAWYFAWLLGTVVIVLVSAAGGVMLDAQIASVGRSEKSDAH-----------
NC_010645-YP_785514-YP_785515-      MAAFFKYIFWLGVSLIAIVLGVLLGDYGPWYLSWVLGTGLMILVAASAGVLFEAQEDAQDKIDAAQGR-----------
                                                                 :*: *: .    :         :
```

Additional File 5

Clade 2 (Light red)

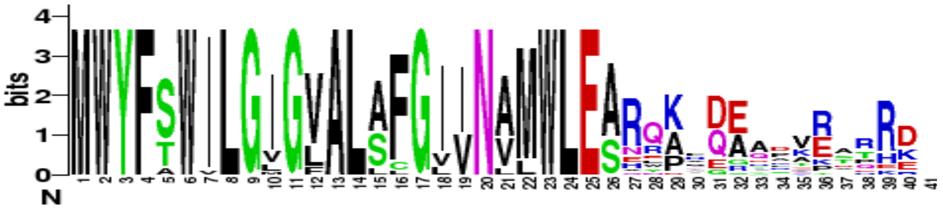

```
NC_012969-YP_003050307-YP_0030      MWYFTWILGVGLALAFGIINVMWLESELPFEEQDTE--------------------
NC_014733-YP_004038974-YP_0040      MWYFTWILGVGLALAFGIINVMWLESELPFEEQDTE--------------------
NC_015856-YP_004753412-YP_0047      MWYFAWILGVGLALAFGIINVMWLEANYAFGSRDEETTRERFESAAAEHEAKARKK
NC_014323-YP_003776453-YP_0037      MWYFAWILGIGLALAFGIINVMWLEANYAFGRRNAEQTHERFASARAAEKARRGGK
NC_010622-YP_001856538-YP_0018      MWYFSWILGIGLALALGIINVMWLEANNAVDAQAARDER-----------------
NC_010804-YP_001947195-YP_0019      MWYFSWILGIGVALSFGIVNAMWLEARQKQQEASVRVPRK----------------
NC_010084-YP_001578676-YP_0015      MWYFSWILGIGVALSFGIVNAMWLEARQKQQEASVRVPRK----------------
NC_008542-YP_836460-YP_836461-      MWYFSWILGIGVALSFGIVNAMWLEARQKTQEAPVRARRD----------------
NC_008060-YP_622077-YP_622078-      MWYFSWILGIGVALSFGIVNAMWLEARQKTQEAPVRARRD----------------
NC_010551-YP_001809428-YP_0018      MWYFSWILGIGVALSFGIVNAMWLEARQKPQEAPVRARRD----------------
NC_008390-YP_774767-YP_774768-      MWYFSWILGIGVALSFGIVNAMWLEARQKPQEAPVRARRD----------------
NC_009256-YP_001120748-YP_0011      MWYFSWILGMGVALSFGIVNAMWLEARQKPQEAAVRTRRD----------------
NC_006348-YP_104657-YP_104656-      MWYFSWILGIGVALAFGIINAMWLEARQKRDA------------------------
NC_008836-YP_001027424-YP_0010      MWYFSWILGIGVALAFGIINAMWLEARQKRDA------------------------
NC_009076-YP_001064844-YP_0010      MWYFSWILGIGVALAFGIINAMWLEARQKRDA------------------------
NC_007651-YP_441012-YP_441011-      MWYFSWVLGIGVALAFGIINAMWLEARQKRDA------------------------
NC_010681-YP_001894217-YP_0018      MWYFSWILGIGVALAFGIINVMWLESRRPEDGSRKTR-------------------
NC_007951-YP_556904-YP_559110-      MWYFSWILGIGVALAFGIINALWLESARPVQGTRRPDQKRV---------------
NC_007951-YP_559109-YP_556903-      MWYFSWILGIGVALAFGIINVMWLESRRPLEAGKPKTH------------------
NC_012724-YP_002912914-YP_0029      MWYFTWILGIGVALSFGVINAMWLESHEAHDAHDA---------------------
NC_015381-YP_004362072-YP_0043      MWYFTWILGIGVALSFGVINAMWLESQEALDAQKQKGQH-----------------
NC_014722-YP_004030295-YP_0040      MWYFSWILGIGVALAFGVINAMWLESHRDAQRLAKPSTEK----------------
NC_008570-YP_856176-YP_856177-      MWYFTWILGLGFALLCGLVNLLWLEARWAADEDLKES-------------------
NC_009348-YP_001142488-YP_0011      MWYFTWILGLGFALLCGLVNLLWLEARWAADEDLKES-------------------
                                    ****:*:**:*.**  *::* :***:
```

Additional File 5

Clade 3 (Maroon)

```
NC_002977-YP_113575-YP_113576       MWYFAWILGVGFACAFGVINAMWLESVCDIDDPAAGGAEN-------
NC_015572-YP_004514181-YP_0045      MWYFAWILGVGFAAAFAIINAMWLESVCDIDTHGIDQSCDTMRKPNS
NC_016112-YP_004915573-YP_0049      MWYFSWILGVGFACAFGIINAMWLEAECDFDHCDMDKAMPGEKCDES
                                    ****:********.**.:*******: **:*    . :
```

Additional File 5

Clade 4 (Dark Red)

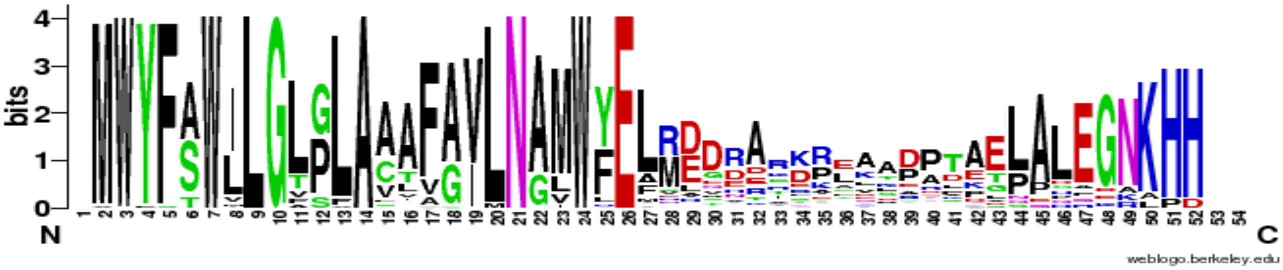


```
NC_017250-YP_005614206-YP_0056      -MWYFSWLLGLPLAAAFAVLNAMWYELMDDRARKRLAADPTAELALEGNKHH--
NC_012442-YP_002734277-YP_0027      -MWYFSWLLGLPLAAAFAVLNAMWYELMDDRARKRLAADPTAELALEGNKHH--
NC_015858-YP_004757703-YP_0047      -MWYFSWLLGLPLAAAFAVLNAMWYELMDDRARKRLAADPTAELALEGNKHH--
NC_010104-YP_001594460-YP_0015      -MWYFSWLLGLPLAAAFAVLNAMWYELMDDRARKRLAADPTAELALEGNKHH--
NC_016796-YP_005152847-YP_0051      -MWYFSWLLGLPLAAAFAVLNAMWYELMDDRARKRLAADPTAELALEGNKHH--
NC_004311-NP_699697-NP_699698-      -MWYFSWLLGLPLAAAFAVLNAMWYELMDDRARKRLAADPTAELALEGNKHH--
NC_013118-YP_003105299-YP_0031      -MWYFSWLLGLPLAAAFAVLNAMWYELMDDRARKRLAADPTAELALEGNKHH--
NC_006933-YP_223479-YP_223478-      -MWYFSWLLGLPLAAAFAVLNAMWYELMDDRARKRLAADPTAELALEGNKHH--
NC_016777-YP_005114314-YP_0051      -MWYFSWLLGLPLAAAFAVLNAMWYELMDDRARKRLAADPTAELALEGNKHH—
NC_009504-YP_001257479-YP_0012      -MWYFSWLLGLPLAAAFAVLNAMWYELMDDRARKRLAADPTAELALEGNKHH--
NC_010167-YP_001622325-YP_0016      -MWYFSWLLGLPLAAAFAVLNAMWYELMDDRARKRLAADPTAELALEGNKHH--
NC_009668-YP_001372076-YP_0013      -MWYFSWLLGLPLAAAFAVLNAMWYELMDDRARKRLAADPTADLAREGKKHH--
NC_011983-YP_002540641-YP_0025      -MWYFAWLLGFPLAAAFAVLNAMWYELVDDNAKKNKRG----------------
NC_003063-NP_356555-2-NP_35655      -MWYFAWLLGLPLAAIFAVMNAMWYELMEENAKKAEAKPEK-------------
NC_015508-YP_004444278-YP_0044      -MWYFAWLLGLPLAAIFAVMNAMWYELMEENARKAEAKPEK-------------
NC_009937-YP_001523500-YP_0015      -MWYFAWILGLPLAAAFAVLNAMWYELMDDEAKKRALTRK--------------
NC_015717-YP_004677510-YP_0046      -MWYFAWLLGLPLAAAFAVLNAMWYELMEDSAAARAQTAPAKGSAQRK------
NC_013446-YP_003279474-YP_0032      -MWYFAWLLGLPLAAAFAVLNAIWFELMDDEATRRKLLERTKSLPDA-------
NC_010002-YP_001563420-YP_0015      -MWYFAWLLGLPLAAAFAVLNAMWYELMDDEATRRELLAREK------------
NC_012791-YP_002945250-YP_0029      -MWYFAWILGLPLAAAFAVLNAMWYELMDDDAIRKEKLALPEAPQGQERL----
NC_009138-YP_001099673-YP_0010      -MWYFAWMLGLPLAAAFAVLNAMWYELMDDEAIRKEKLGNS-------------
NC_009659-YP_001353704-YP_0013      -MWYFSWMLGLPLAAAFAVLNAMWYELMDDEAIRKEKLNKPS------------
NC_014625-YP_003962821-YP_0039      -MWYFAWILGLPLAVTFAVLNAMWFELVDDAKAKKPEADAPDL-----------
NC_017384-YP_005796367-YP_0057      -MWYFAWILGLPLAVTFAVLNAMWFELVDDAKAKKPEADAPDL-----------
NC_011420-YP_002300189-YP_0023      -MWYFAWLLGLPLAVVFAVLNGLWFELREDAARGQPDPDAF-------------
NC_014034-YP_003579216-YP_0035      -MWYFAWLLGLPVAVMFGVLNAMWFEMQEDRRRDGEV-----------------
NC_012988-YP_003067568-YP_0030      -MWYFAWILGLGLAAAVGVLNALWYELRAVRETPPEVTPPLPTP----------
NC_012808-YP_002962309-YP_0029      -MWYFAWILGLGLAAAVGVLNALWYELRAVRETPPEVTPPLPTP----------
NC_011757-YP_002420353-YP_0024      -MWYFAWILGLGLAAAVGVLNALWYELRAVRETPPEVTPPLPTP----------
NC_010725-YP_001924011-YP_0019      -MWYFAWILGLGLAAAVGVLNALWYELRAVRETPPEVTPTLPTP----------
NC_011527-YP_002303533-YP_0023      MMWYFAWILGFGLACAFAILNALWFELERYKDKS--------------------
NC_011528-YP_002305253-YP_0023      MMWYFAWILGFGLACAFAILNALWFELERYKDKS--------------------
NC_016147-YP_004931548-YP_0049      -MWYFAWILGVSLACFFAILNGMWFEMRQDARKGVAPDPQTQGNPPPDAAPDEL
NC_016078-YP_004900063-YP_0049      -MWYFSWILGVGLACAFAILNAMWFEMREDRREMRDKS----------------
NC_015947-YP_004793274-YP_0047      -MWYFAWILGAGLASTVAILNGMWFEAREQNRIEKENRR---------------
NC_017671-YP_006185556-YP_0061      -MWYFAWILGAGLASTVAILNGMWFEAREQNRIEKENRR---------------
NC_003902-NP_637588-NP_637589-      -MWYFAWILGTGLAALAAVLNGMWFEAREQDTPH--------------------
NC_007086-YP_242968-YP_242967-      -MWYSAWILGTGLAALAAVLNGMWFEAREQDTPH--------------------
NC_003919-NP_642653-NP_642654-      -MWYFAWILGTGLAALAAVLNGMWFEAREPERGEPSQ-----------------
NC_016010-YP_004851905-YP_0048      -MWYFAWILGTGLAALAAVLNGMWFEAREPERGEPPQ-----------------
NC_017223-YP_005588539-YP_0055      -MWYFSWILGLSLACAFGILNAMWFELREGHAHDPYKKPADE------------
NC_002927-NP_891032-NP_891031-      -MWFFSWILGLSLACAFGILNAMWFELREGHAHDPYKKPADE------------
NC_002929-NP_879142-NP_879143-      -MWYFSWILGLSLACAFGILNAMWFELREGHAHDPYKKPADE------------
NC_002928-NP_886170-NP_886169-      -MWYFSWILGLSLACAFGILNAMWFELREGHAHDPYKKPADE------------
NC_010170-YP_001629059-YP_0016      -MWYFSWILGLSLACAFGILNAMWFELREGGEHDPARPGGA-------------
NC_014640-YP_003982142-YP_0039      -MWYFSWILGLGLACAFAILNAMWFELREGHTHDPRASRNDA------------
NC_010645-YP_787606-YP_787605-      -MWYFSWILGLGLACTFAILNAMWFELREGQAHDPLKQPVSK------------
NC_014125-YP_003618534-YP_0036      -MWYFSWILGTGLACVFAVLNAVWLELREEDVKQ--------------------
NC_009494-YP_001249996-YP_0012      -MWYFSWILGTGLACVFAVLNAVWLEIREEDDKQ--------------------
NC_013861-YP_003456054-YP_0034      -VWYFSWILGTGLACCFAVLNAMWLELRDSDTESSNKDSR--------------
NC_013854-YP_003447980-YP_0034      -MWYFAWVLGVGFAFACGILNAVWHEHHLP----ATDTVD--------------
NC_014414-YP_003855869-YP_0038      -MWYFAWALGLGFAALVGILNAMWFELKRTRQADGTESAPILGQEH--------
NC_008358-YP_760367-YP_760366-      -MWYFTWILGLGFALTFSILNAMWNEVSMGEAGENDLEIDR-------------
NC_009511-YP_001264219-YP_0012      -MWYFSWVLGVGLAVGFGILNGMWHEFHSDPDDDAAA-----------------
NC_014816-YP_004086755-YP_0040      -MWYFTWVLGLGLAVTFGILNGIWFEFLTTDDPDAEDPHRD-------------
NC_010338-YP_001682265-YP_0016      -MWYFTWILGLGLALAFGVLNGVWYEFNLTDDGDLGVSEDT-------------
NC_014100-YP_003594646-YP_0035      -MWYFTWILGLGLAVAFGVLNGVWYEFNLADDGDEGLSEP--------------
NC_002696-NP_419579-NP_419580-      -MWYFTWILGLGLAVAFGVLNGMWYEFSLSDEGDEGLDEA--------------
NC_011916-YP_002516173-YP_0025      -MWYFTWILGLGLAVAFGVLNGMWYEFSLSDEGDEGLDEA--------------
                                      :*: :* **  .*   .::*.:* *
```

Additional File 5

<u>Clade 5 (Yellow)</u>

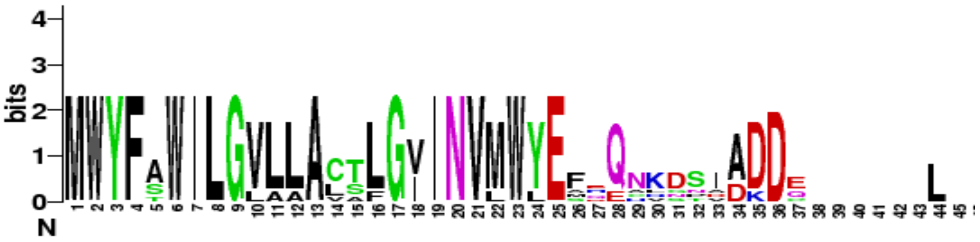

```
NC_007481-YP_340517-YP_340518-      MWYFAWILGVLLACTLGVINVMWYEFQQNKDSIADDEQVMHDLLKETNTTKNDD
NC_014803-YP_004069149-YP_0040      MWYFAWILGVLLACTLGVINVMWYEFHQNKNSIADDEQATHDLLNEHNTTHNDD
NC_015497-YP_004435143-YP_0044      MWYFAWILGVLLACTLGVINVMWYEFQQNKDSIADDQKELYEKLQKERDN----
NC_008228-YP_661029-YP_661030-      MWYFAWILGVLLACTLGVINVMWYEFHQNKDSIADDEKELYERLQKERDN----
NC_013851-YP_003444443-YP_0034      MWYFSWILGVLLALAFGIINVLWYESDQCRSTADDDCPT---------------
NC_003910-YP_268606-YP_268607-      MWYFSWILGVLLAVSLGIINVMWYEMEQHVDNVAKDQDTDDQA-----------
NC_017059-YP_005416849-YP_0054      MWYFTWILGLAAALSLGIINVMWLEAEEALGPDDD-------------------
                                    ****:****:  * ::*:***:* * .:  .   .
```

Clade 6 (Orange)

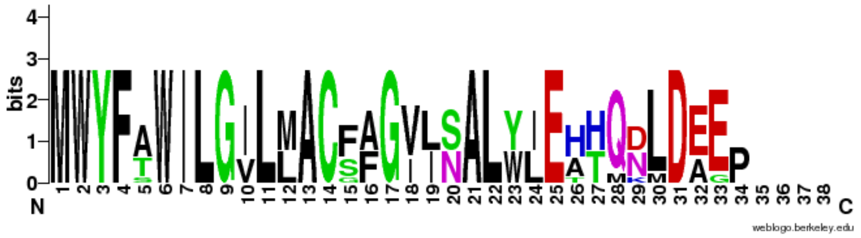


```
NC_011586-YP_002319615-YP_0023      MWYFAWILGILMACFAGVLSALYIEHHQNLDEE-----
NC_014259-YP_003731902-YP_0037      MWYFAWILGILMACFAGVLSALYIEHHQNLDEE-----
NC_017162-YP_005515077-YP_0055      MWYFAWILGILMACFAGVLSALYIEHHQNLDEE-----
NC_005966-YP_046898-YP_046899-      MWYFAWILGILMACFAGVLSALYIEHHQNLDEE-----
NC_016603-YP_004995673-YP_0049      MWYFAWILGILMACFAGVLSALYIEHHQDLDEE-----
NC_015740-YP_004713644-YP_0047      MWYFTWILGVLLACSFGIINALWLEATQDLDAEP----
NC_017532-YP_005937964-YP_0059      MWYFTWILGVLLACSFGIINALWLEATQDLDAEP----
NC_009434-YP_001171817-YP_0011      MWYFTWILGVLLACSFGIINALWLEATQDLDAEP----
NC_012560-YP_002799172-YP_0027      MWYFSWILGILLACGFGVINALWLETTMKMDEGPAGGE
                                    ****:****:*:**  *::.**::*   .:*
```

Clade 7 (Pea Green)

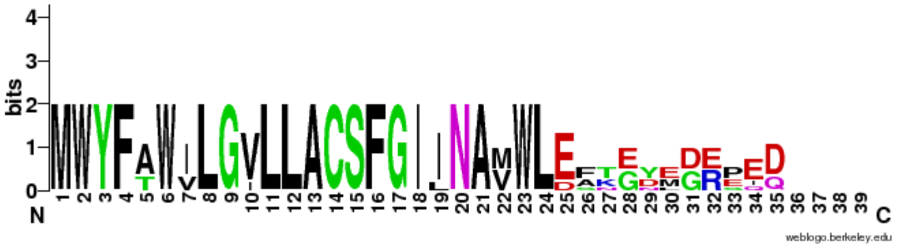


```
NC_012997-YP_003075320-YP_0030      MWYFTWILGVLLACSFGIINAMWLEATEDLDRSIDESEG
NC_015554-YP_004469330-YP_0044      MWYFTWILGVLLACSFGILNAMWLESTEDMDRPEDAE--
NC_011138-YP_004428935-YP_0044      MWYFAWILGVLLACSFGIINAMWLEATENMDRPEDQE--
NC_009654-YP_001340368-YP_0013      MWYFAWVLGVLLACSFGIINAVWLEFKGYEGEEEQK---
NC_015559-YP_004482249-YP_0044      MWYFAWILGILLACSFGIINAVWLEFNGYEGEEEQH---
NC_015276-YP_004313696-YP_0043      MWYFAWVLGVLLACSFGIINAVWLDFKGYEGEPQD----
                                    ****:*:**:********:**:**: .   ..  :
```

Clade 8 (Light Green)

NC_008709-YP_942171-YP_942172-YP_942173   MWYLAWMLGVLLACSLGIINALWYEQVEANENAEGIDEDQHS

<u>Clade 9 (Dark Green)</u>

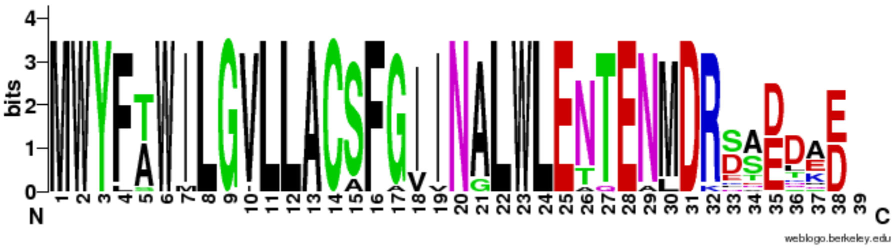


```
NC_008321-YP_733376-YP_733377-       MWYFTWILGVLLACSFGVINALWLENTENMDRSSDDAE-
NC_008577-YP_868882-YP_868883-       MWYFTWILGVLLACSFGVINALWLENTENMDRSSDDAE-
NC_004347-NP_718840-NP_718839-       MWYFTWILGVLLACAFGVINALWLENTENMDRSSDDPE-
NC_017566-YP_006010650-YP_0060       MWYFTWILGVLLACSFGIINALWLENTENMDRSAEDAE-
NC_009438-YP_001184161-YP_0011       MWYFTWILGVLLACSFGIINALWLENTENMDRSAEDAE-
NC_017571-YP_006019951-YP_0060       MWYFTWILGVLLACSFGIINALWLENTENMDRSADDAE-
NC_017579-YP_006038300-YP_0060       MWYFTWILGVLLACSFGIINALWLENTENMDRSADDAE-
NC_008700-YP_928224-YP_928223-       MWYFTWILGVLLACSFGIINALWLENTENMDRKSDDE--
NC_007954-YP_562283-YP_562284-       MWYFAWILGVLLACSFGIINALWLENTENLDRLSDDSED
NC_014541-YP_003913971-YP_0039       MWYFAWILGVLLACAFGIINGLWLEAQEALDKDED----
NC_009831-YP_001474850-YP_0014       MWYFAWILGVLLACSFGIINALWLENTENMDREADKQD-
NC_011566-YP_002310842-YP_0023       MWYFAWILGVLLACSFGIINALWLENTENMDREVESED-
NC_008345-YP_749833-YP_749834-       MWYFAWILGVLLACSFGIINALWLENTENMDRNNEL---
NC_009092-YP_001093431-YP_0010       MWYFAWILGILLACSFGIINALWLENTENMDRDSELKD-
NC_010506-YP_001759946-YP_0017       MWYFAWILGVLLACSFGIINALWLENTENMDRDTENKD-
NC_009901-YP_001501180-YP_0015       MWYFAWILGVLLACSFGIINALWLETTENMDRDAETED-
NC_010334-YP_001673607-YP_0016       MWYFSWILGVLLACSFGIINALWLETTENMDRDAETED-
NC_014012-YP_003556137-YP_0035       MWYLTWMLGVLLACSFAIVNGLWLETTENMDRDTEQKD-
                                     ***::*:**:****:*.::*.****  * :*:  :
```

Additional File 5

Clade 10 (Light Blue)

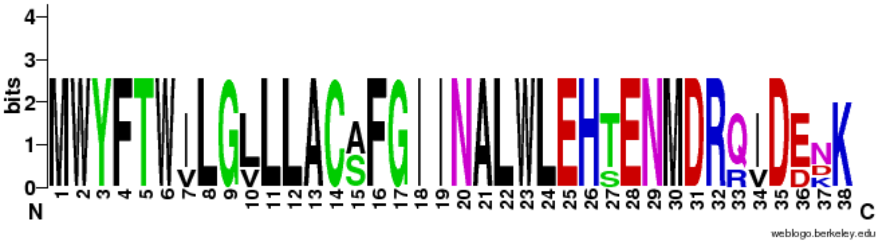


```
NC_015424-YP_004391251-YP_0043      MWYFTWILGLLLACAFGIINALWLEHTENMDRQIDENK
NC_008570-YP_858102-YP_858101-      MWYFTWILGLLLACAFGIINALWLEHTENMDRQIDENK
NC_009348-YP_001140647-YP_0011      MWYFTWILGLLLACSFGIINALWLEHSENMDRQIDEDK
NC_012691-YP_002893889-YP_0028      MWYFTWVLGVLLACSFGIINALWLEHTENMDRRVDDK-
                                    ******:**:****:***********:*****::*:.
```

Clade 11 (Dark Blue)

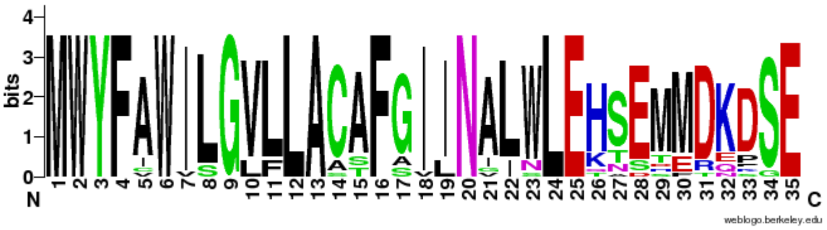


```
NC_013456-YP_003286546-YP_0032      MWYFAWILGVLLACAFGIINALWLEHSEMMDKDSE
NC_016602-YP_004993493-YP_0049      MWYFAWILGVLLACAFGIINALWLEHSEMMDKDSE
NC_004603-NP_797432-NP_797433-      MWYFAWILGVLLACAFGIINALWLEHSEMMDKDSE
NC_016613-YP_005022451-YP_0050      MWYFAWILGVLLACAFGIINALWLEHSEMMDKDSE
NC_009783-YP_001444799-YP_0014      MWYFAWILGVLLACAFGIINALWLEHSEMMDKDSE
NC_015633-YP_004565937-YP_0045      MWYFAWILGVLLACAFGIINALWLEHSEMMDKDSE
NC_014965-YP_004188254-YP_0041      MWYFAWILGVLLACAFGIINALWLEHSEMMDKDSE
NC_004459-NP_761020-2-NP_76102      MWYFAWILGVLLACAFGIINALWLEHSEMMDKDSE
NC_011753-YP_002416724-YP_0024      MWYFAWILGVLLACAFGIINALWLEHSEMMDKDSE
NC_011184-YP_002155716-YP_0021      MWYFAWILGVLLACAFGIINALWLEHSEMMDKDSE
NC_006840-YP_204336-YP_204337-      MWYFAWILGVLLACAFGIINALWLEHSEMMDKDSE
NC_017270-YP_005634201-YP_0056      MWYFAWILGVLLACAFGIINALWLEHTEMMDKDSE
NC_016944-YP_005333649-YP_0053      MWYFAWILGVLLACAFGIINALWLEHTEMMDKDSE
NC_011312-YP_002263286-YP_0022      MWYFAWILGVLLACSFAIINALWLEHSEMMDKDSE
NC_006370-YP_130739-YP_130738-      MWYFAWILGVLLACSFGIINALWLETTEDMDNKGE
NC_015633-YP_004566093-YP_0045      MWYFCWVLGVLLACAFGIINGLWLEQAEHFDES--
NC_017269-YP_005632513-YP_0056      MWYFIWISGLFLAATFSILNIINLEKNSTERQP--
NC_016945-YP_005335319-YP_0053      MWYFIWISGLFLAATFSILNIINLEKNSTERQP--
NC_011753-YP_002417300-YP_0024      MWYFVWILGLFLASAFAVLNVISLEKSDSETEF--
                                    **** *: *::**.:*.::* : **  .   :
```

Clade 12 (Light Purple)

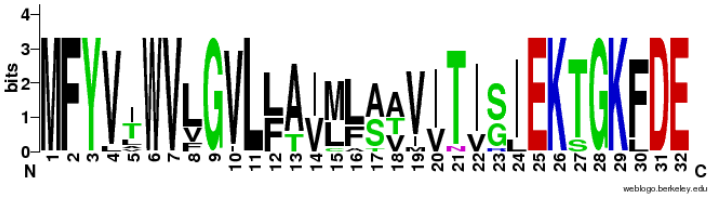


```
NC_009053-YP_001053006-YP_0010      MFYVTWVLGVLLAILFATVITVSIEKTGKFDE
NC_011852-YP_002474719-YP_0024      MFYVTWVLGVLLAILFATVITVSIEKTGKFDE
NC_010278-YP_001651345-YP_0016      MFYVTWVLGVLLAILFATVITVSIEKTGKFDE
NC_002940-NP_874145-NP_874144-      MFYVTWVLGILLAILFATVVTIGIEKSGKFDE
NC_006300-YP_087907-YP_087908-      MFYLAWVVGVLLAILASVMITIRIEKSGKFDE
NC_016513-YP_004947818-YP_0049      MFYVIWVFGVLLAIMLSVVVTISIEKTGKFDE
NC_013416-YP_003256421-YP_0032      MFYVIWVFGVLLAIMLSVVVTISIEKTGKFDE
NC_012913-YP_003007789-YP_0030      MFYVVWVLGVLLAIMLSVVVTISIEKTGKFDE
NC_017027-YP_005362685-YP_0053      MFYVIWVLGVLFTVMLAAVITIGIEKTGKFDE
NC_002663-NP_245911-NP_245910-      MFYVIWVLGVLFTVMLAAVITIGIEKTGKFDE
NC_016808-YP_005176115-YP_0051      MFYVIWVLGVLFTVMLAAVITIGIEKTGKFDE
NC_008309-YP_718464-YP_718465-      MFYVLWVVGVLFAVMLSAIITIGLEKTGKLDE
NC_010519-YP_001783482-YP_0017      MFYVLWVVGVLFAVMLSAIITISLEKTGKLDE
NC_015460-YP_004420951-YP_0044      MFYVTWVLGVLFAVCLTAVINISIEKTGKFDE
                                    ***: **.*:*:::  :.::.: :**:**:**
```

Clade 13 (Dark Purple)

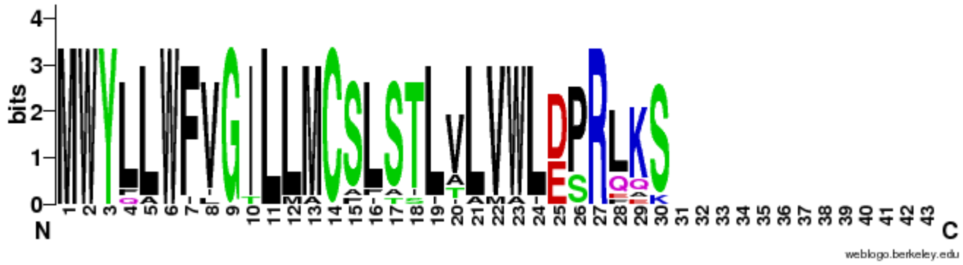


```
NC_010658-YP_001880824-YP_0018      MWYLLWFVGILLMCSLSTLVLVWLDPRLKS-------------
NC_007613-YP_408646-YP_408645-      MWYLLWFVGILLMCSLSTLVLVWLDPRLKS-------------
NC_016822-YP_005455632-YP_0054      MWYLLWFVGILLMCSLSTLVLVWLDPRLKS-------------
NC_007384-YP_309953-YP_309954-      MWYLLWFVGILLMCSLSTLVLVWLDPRLKS-------------
NC_011748-YP_002402178-YP_0024      MWYLLWFVGILLMCSLSTLVLVWLDPRLKS-------------
NC_008253-YP_668897-YP_668898-      MWYLLWFVGILLMCSLSTLVLVWLDPRLKS-------------
NC_004741-NP_836687-NP_836688-      MWYLLWFVGILLMCSLSTLVLVWLDPRLKS-------------
NC_017328-YP_005726619-YP_0057      MWYLLWFVGILLMCSLSTLVLVWLDPRLKS-------------
NC_007606-YP_402616-YP_402617-      MWYLLWFIGILLMCSLSTLVLVWLDPRLKS-------------
NC_013508-YP_003297038-YP_0032      MWYLLWFVGILLMCSLSTLILVWLEPRLK--------------
NC_010067-YP_001570193-YP_0015      MWYLLWFVGILLMCSLSTLALVWLESRQQ--------------
NC_011149-YP_002146232-YP_0021      MWYLLWFVGILLMCSLSTLALVWLESRQQ--------------
NC_015761-YP_004730506-YP_0047      MWYLLWFVGILLMCSLSTLTLVWLESRQK--------------
NC_011740-YP_002382344-YP_0023      MWYQLWFVGILLMCAITSLVLVWLEPRFESAKKDEADGNHKIA
NC_012779-YP_002934774-YP_0029      MWYFAWILGTLMACFFAIITAMAIESREAKAAQDGK-------
                                    ***  *::* *: * :: :  : ::.*
```

Clade 14 (Black)

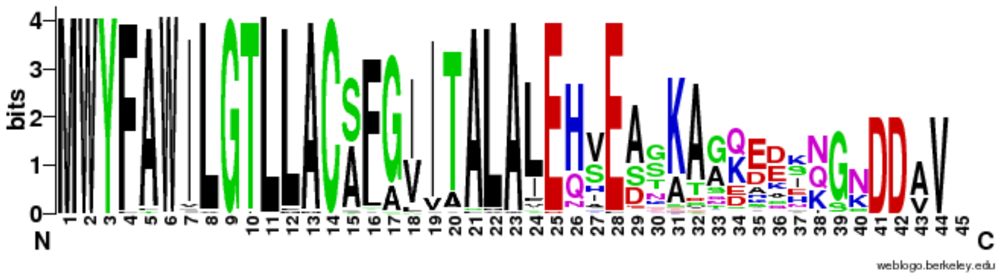


```
NC_011748-YP_002401824-YP_0024      MWYFAWILGTLLACSFGVITALALEHVESGKAGQEDI--------
NC_008253-YP_668668-YP_668669-      MWYFAWILGTLLACSFGVITALALEHVESGKAGQEDI--------
NC_010658-YP_001879389-YP_0018      MWYFAWILGTLLACSFGVITALALEHVESGKAGQEDI--------
NC_016822-YP_005455310-YP_0054      MWYFAWILGTLLACSFGVITALALEHVESGKAGQEDI--------
NC_007384-YP_309676-YP_309677-      MWYFAWILGTLLACSFGVITALALEHVESGKAGQEDI--------
NC_017328-YP_005726229-YP_0057      MWYFAWILGTLLACSFGVITALALEHVESGKAGQEDI--------
NC_004741-NP_836275-NP_836274-      MWYFAWILGTLLACSFGVITALALEHVESGKAGQEDI--------
NC_007613-YP_407107-YP_407108-      MWYFAWILGTLLACSFGVITALALEHVESGKAGQEDI--------
NC_007606-YP_402357-YP_402358-      MWYFAWILGTLLACSFGVITALALEHVESGKAGQEDI--------
NC_011740-YP_002383485-YP_0023      MWYFAWILGTLLACSFGVITALALEHVESGKAGQEDI--------
NC_009792-YP_001453963-YP_0014      MWYFAWILGTLLACAFGIITALALEHVESGKAGQEDS--------
NC_009778-YP_001438690-YP_0014      MWYFAWILGTLLACAFGIITALALEHVEATKAGKVES--------
NC_013282-YP_003209702-YP_0032      MWYFAWILGTLLACAFGIITALALEHVEATKAGKVES--------
NC_016612-YP_005018900-YP_0050      MWYFAWILGTLLACAFGVITALALEHVEATKAGKEEH--------
NC_015663-YP_004593031-YP_0045      MWYFAWILGTLLACAFGVITALALEHVEATKAGKEEH--------
NC_013850-YP_003440546-YP_0034      MWYFAWILGTLLACAFGVITALALEHVEATKAGKEEH--------
NC_011283-YP_002239646-YP_0022      MWYFAWILGTLLACAFGVITALALEHVEATKAGKEEH--------
NC_017540-YP_005953790-YP_0059      MWYFAWILGTLLACAFGVITALALEHVEATKAGKKEEI-------
NC_011149-YP_002145701-YP_0021      MWYFAWILGTLLACAFGIITALALEHVEAGKTGQEES--------
NC_015761-YP_004729534-YP_0047      MWYFAWILGTLLACAFGIITALALEHVEAGKTGQEES--------
NC_010067-YP_001571210-YP_0015      MWYFAWILGTLLACAFGIITALALEHVEADKTGQKES--------
NC_013716-YP_003364350-YP_0033      MWYFAWILGTLLACAFGIITALALEHVEASKAGQEEH--------
NC_016514-YP_004951141-YP_0049      MWYFAWILGTLLACAFGVITALALEHVEASKAGEDKH--------
NC_012962-YP_003039711-YP_0030      MWYFAWVLGTLLACAVAIVVALASDD-------------------
NC_010694-YP_001908224-YP_0019      MWYFAWVLGTLLACGCGIITALALEQAEAQSGSNEKS--------
NC_014562-YP_003930206-YP_0039      MWYFAWILGTLLACAFGIVTALAIEHVEAQDETH-----------
NC_015735-YP_004706727-YP_0047      MWYFTWILGTLLACALGIITALAIEHLENTSSG------------
NC_007712-YP_454560-YP_454561-      MWYFAWILGTLLACAFGIITALALEHLEDTRGEDAMK--------
NC_016816-YP_005196538-YP_0051      MWYFAWILGTLLACAFGIITALAIEHIEDSAASDRQS--------
NC_017554-YP_005993696-YP_0059      MWYFAWILGTLLACAFGIITALAIEHIEDSAASDRQS--------
NC_013956-YP_003519475-YP_0035      MWYFAWILGTLLACAFGIITALAIEHIEDSAASDRQS--------
NC_017531-YP_005933377-YP_0059      MWYFAWILGTLLACAFGIITALAIEHIEDSAASDRQS--------
NC_014306-YP_003740685-YP_0037      MWYFAWILGTLLACAFGIITALAIEHIEDSEVKEKR---------
NC_015968-YP_004827742-YP_0048      MWYFAWILGTLLACAFGIITALALEHIEASKASDEKH--------
NC_015566-YP_004499691-YP_0044      MWYFAWILGTLLACAFGIITALALEHHEDSKAQQDGK--------
NC_017573-YP_006024105-YP_0060      MWYFAWILGTLLACAFGIITALALEHHEDSKAQQDGK--------
NC_015567-YP_004504643-YP_0045      MWYFAWILGTLLACAFGIITALALEHHEDSKAQQDGK--------
NC_009832-YP_001477503-YP_0014      MWYFAWILGTLLACAFGIITALALEHHEDSKAQQDDK--------
NC_016818-YP_005201131-YP_0052      MWYFAWILGTLLACSFGIITALAFEHSEANKAAKEEK--------
NC_015061-YP_004213866-YP_0042      MWYFAWILGTLLACSFGIITALAFEHSEANKAAKEEK--------
NC_017047-YP_005402952-YP_0054      MWYFAWILGTLLACSFGIITALAFEHSEANKAAKEEK--------
NC_008800-YP_001007127-YP_0010      MWYFAWILGTLLACSFGIITALALEQSEANAAAKAANNGKDDVVR
NC_015224-YP_004297499-YP_0042      MWYFAWILGTLLACSFGIITALALEQSEANAAAKAANNGKDDVVR
NC_009708-YP_001401839-YP_0014      MWYFAWILGTLLACSFGIITALALEQSEANAAAKAANNGNDDAV-
NC_010159-YP_001605914-YP_0016      MWYFAWILGTLLACSFGIITALALEQSEANAAAKAANNGNDDAV-
NC_017168-YP_005523522-YP_0055      MWYFAWILGTLLACSFGIITALALEQSEANAAAKAANNGNDDAV-
NC_006155-YP_069686-YP_069687-      MWYFAWILGTLLACSFGIITALALEQSEANAAAKAANNGNDDAV-
NC_014500-YP_003882117-YP_0038      MWYFAWILGTLLACSLGVITALALEQSEATKTNQDKPQ-------
NC_012912-YP_003005211-YP_0030      MWYFAWILGTLLACSLGVITALALEQSEATKTNQDKPQ-------
NC_013592-YP_003332781-YP_0033      MWYFAWILGTLLACSLGVITALALEQSEATKTNKDKL--------
NC_013421-YP_003260444-YP_0032      MWYFAWILGTLLACSLGIITALAIEQSEASKAVEDDKQ-------
NC_004547-YP_049471-YP_049472-      MWYFAWILGTLLACSLGIITALAIEQSEASKAAEEDKQ-------
NC_012917-YP_003016825-YP_0030      MWYFAWILGTLLACSLGIITALALEQSEASKAAEEDKQ-------
NC_012917-YP_003018304-YP_0030      MWYFAWILGTLLACAFALVTALAVENHEAGKDAS-----------
NC_004547-YP_051089-YP_051090-      MWYFAWILGTLLACAFALVTALAVENHEAGKDAS-----------
NC_013421-YP_003258939-YP_0032      MWYFAWILGTLLACAFALVTALAVENHEAGKDA------------
NC_013892-YP_003466984-YP_0034      MWYFAWILGTLLACSFAIIAALALEQSESKKATESGEK-------
NC_014228-YP_003711676-YP_0037      MWYFAWILGTLLACSFAIIAALALEHSESKKATESDEK-------
NC_005126-NP_928745-NP_928746-      MWYFAWILGTLLACSFAIIAALALEHNEEEKVAKNSEKSC-----
NC_012962-YP_003041797-YP_0030      MWYFAWILGTLLACSFAIIAALALEHHEAEKAAENGEK-------
NC_010554-YP_002150342-YP_0021      MWYFAWLLGTLFACSIAVIAGLAYEHAEAKKTSESEE--------
NC_017731-YP_006217662-YP_0062      MWYFAWILGTLLACSFAVIAALALEHAEDKAAAKDGE--------
NC_014837-YP_004114997-YP_0041      MWYFAWILGTLLACSFGVIAALALERAEEAAAKGDKA--------
NC_013508-YP_003296626-YP_0032      MWYFAWILGTLMACFFAIITAMAIENRETKAAQDGK---------
NC_012779-YP_002934260-YP_0029      MWYLLWFVGILLMCSLSTLILVWLEPRLK----------------
                                    ***: *.:* *: *   . :   :   :
```

Additional File 5
